# The Effect of a Diiodothyronine Mimetic on Insulin Sensitivity in Male Cardiometabolic Patients: A Double-Blind Randomized Controlled Trial

**DOI:** 10.1371/journal.pone.0086890

**Published:** 2014-02-21

**Authors:** Fleur van der Valk, Carlijne Hassing, Maartje Visser, Purav Thakkar, Anookh Mohanan, Kaushal Pathak, Chaitanya Dutt, Vijay Chauthaiwale, Mariette Ackermans, Aart Nederveen, Mireille Serlie, Max Nieuwdorp, Erik Stroes

**Affiliations:** 1 Department of Vascular Medicine, Academic Medical Center, Amsterdam, the Netherlands; 2 Clinical Research Department, Torrent Pharmaceuticals Limited, Village-Bhat, Dist. Gandhinagar, India; 3 Department of Clinical Chemistry, Laboratory of Endocrinology, Academic Medical Center, Amsterdam, the Netherlands; 4 Department of Radiology, Academic Medical Center, Amsterdam, the Netherlands; 5 Department of Endocrinology, Academic Medical Center, Amsterdam, the Netherlands; Postgraduate Medical Institute & Hull York Medical School, University of Hull, United Kingdom

## Abstract

**Background and aims:**

Obesity and its associated cardiometabolic co-morbidities are increasing worldwide. Since thyroid hormone mimetics are capable of uncoupling the beneficial metabolic effects of thyroid hormones from their deleterious effects on heart, bone and muscle, this class of drug is considered as adjacent therapeutics to weight-lowering strategies. This study investigated the safety and efficacy of TRC150094, a thyroid hormone mimetic.

**Materials and Methods:**

This 4-week, randomized, placebo-controlled, double-blind trial was conducted in India and The Netherlands. Forty subjects were randomized at a 1∶1 ratio to receive either TRC150094 dosed at 50 mg or placebo once daily for 4 weeks. Hyperinsulinemic euglycemic clamp and ^1^H-Magnetic Resonance Spectroscopy (MRS) were performed before and after treatment.

**Results:**

At baseline, subjects were characterized by markedly impaired hepatic and peripheral insulin sensitivity. TRC150094 dosed 50 mg once daily was safe and well tolerated. Hepatic nor peripheral insulin sensitivity improved after TRC150094 treatment, expressed as the suppression of Endogenous Glucose Production from 59.5 to 62.1%; p = 0.477, and the rate of glucose disappearance from 28.8 to 26.4 µmol kg^−1^min^−1^, p = 0.185. TRC150094 administration did not result in differences in fasting plasma free fatty acids from 0.51 to 0.51 mmol/L, p = 0.887 or in insulin-mediated suppression of lipolysis from 57 to 54%, p = 0.102. Also, intrahepatic triglyceride content was unaltered.

**Conclusion:**

Collectively, these data show that, in contrast to the potent metabolic effects in experimental models, TRC150094 at a dose of 50 mg daily does not improve the metabolic homeostasis in subjects at an increased cardiometabolic risk. Further studies are needed to evaluate whether TRC150094 has beneficial effects in patients with more severe metabolic derangement, such as overt diabetes mellitus and hypertriglyceridemia.

**Trial Registration:**

clinicaltrials.gov NCT01408667

## Introduction

Obesity and its associated co-morbidities have a detrimental impact on global health, representing the leading cause of disability and premature death worldwide. [1]. Hence, there is an unmet medical need for safe and effective therapies aimed at preventing the cardiometabolic sequelae associated with central adiposity. Among the potential candidates, thyroid hormones (TH) have been shown to increase basal energy expenditure and oxygen consumption leading to favorable improvements in lipid and glucose metabolism. [2], [3] In a clinical setting, however, TH have failed predominantly due to cardiotoxicity, as well as bone and muscle toxicity. [4] Subsequently, selective TH analogs were designed in an effort to retain the beneficial effects whilst avoiding the toxic side effects. Analogs of TH with a 22-fold higher affinity for the hepatic thyroid hormone receptor beta (TRβ) than the ‘ubiquitous’ TRα isoform were reported to lower low density lipoprotein cholesterol (LDLc) by approximately 30% without significant heart, muscle or bone toxicity. [5] Though promising [5], further development had to be discontinued due to the observation of increased cartilage damage following prolonged exposure to these TH analogs in dogs. [6].

More recently, another way to circumvent the negative effects of TH, a mimetic of diiodothyronine (T2) - TRC150094– was studied in a phase I study (data not published). TRC150094 has a very low potency for both TR isoforms when compared to TH such as T3. Therefore, the mechanism of action of T2 has been attributed to a direct, receptor-independent interaction of T2 with mitochondria. [7] In preclinical studies, TRC150094 was shown to stimulate mitochondrial fatty acid oxidation (FAO) which led to a reduction of visceral adiposity in Wistar rats. [8] In line, TRC150094 improved glucose tolerance and hepatic steatosis in obese Zucker spontaneously hypertensive fatty (ZSF1) rats with a concomitant reduction in plasma cholesterol and triglycerides in ZSF1 rats. [9], [10] Most importantly, TRC150094 was not associated with any adverse safety signal in experimental models up to 24 weeks. [8], [9] In phase I clinical studies, once daily oral administration of TRC150094 at doses of 50 mg and 150 mg for 28 days were well tolerated without any adverse safety signals in the obese subjects.

In the present study, we set out to evaluate the safety and the effect of TRC150094 on insulin sensitivity, liver fat content and lipid profile in obese male subjects with an increased cardiometabolic risk.

## Methods

### Study Design

This randomized, placebo-controlled, double-blind trial was conducted at 2 sites and was approved by the local Institutional Review board at Veeda Clinical Research, India and at the Academic Medical Centre (AMC), The Netherlands. The trial was conducted according to the principles of the International Conference on Harmonisation–Good Clinical Practice guidelines, and externally monitored by an independent contract research organization and registered on clinicaltrials.gov (NCT01408667). All participants provided written informed consent. In total, 40 subjects were enrolled; 20 subjects at Veeda Clinical Research, Ahmedabad, India, and 20 subjects at AMC, Amsterdam, The Netherlands. Each subject attended the study center for 5 visits; 1 screening visit, 2 study visits (1 baseline and 1 end of treatment), 1 intermediate safety visit and 1 post-study follow-up visit. During all 5 visits physical examination, vital signs, safety biochemistry and laboratory investigations were performed and evaluated by a physician blinded for treatment allocation. The protocol for this trial and supporting CONSORT checklist are available as supporting information file S1; see Checklist S1 and Protocol S1.

### Outcome Measures

Before and after treatment a hyperinsulinemic euglycemic clamp and ^1^H-Magnetic Resonance Spectroscopy (MRS) were performed. In brief, the clamp technique is a method to evaluate insulin sensitivity in which insulin is infused in two dose steps and plasma glucose concentration is held constant by a variable glucose infusion. Hepatic insulin sensitivity is assessed with a low dose insulin infusion, suppressing the endogenous glucose production (EGP). Subsequently, peripheral insulin sensitivity is assessed via high dose insulin infusion, measuring the rate of glucose disposal (Rd). Intrahepatic triglyceride content was assessed via MRS spectra acquired on a 3.0 T Intera scanner (Philips, Best, the Netherlands). More detailed information on study procedures is provided in File S1. Primary efficacy variable was the change in insulin sensitivity from baseline to week 4. Secondary efficacy variables were changes in hepatic fat content (IHTG) and lipid profile. Safety assessments included documentation of adverse events, blood pressure, heart rate, body temperature, weight and laboratory tests, including thyroid and liver-function tests.

### Patient Selection

Eligible subjects were male, aged 30 to 65 years, and characterized by a metabolic syndrome when meeting all of the following criteria [11]: increased waist circumference (Indian descent ≥90 cm, Western European descent ≥102 cm), blood pressure ≥130/85 mmHg or use of antihypertensive drugs, fasting glucose >5.5 mmol/l–11.0 mmol/l and fasting insulin level ≥10 mU/mL. Subjects were considered not eligible in case of history of somatic illness, including neoplasm, endocrine or neurologic disorders, active infection, unstable weight 3 months prior to inclusion or recent surgical procedure within 3 months of the study initiation; respectively systolic and diastolic blood pressure of ≥160 mmhg or ≥100 mmHg, impaired kidney function (eGFR <60 mL/min/1.73 m2 as evaluated by MDRD method) or impaired liver function (ALT or AST >3×ULN) at screening.

### Randomisation and Blinding

The randomization procedure was handled by the clinical research unit at each site. A randomisation code was made in consecutive sequence at the two study centres and specified whether the subject receives TRC150094 or placebo dosing over the course of the study period. All randomisation information was secured and kept in a locked storage area, accessible only by authorised personnel. After screening, subjects were randomized at allocation in a 1∶1 ratio to receive either TRC150094 dosed at 50 mg or placebo once daily for 28 days. The placebo tablets were identical to the TRC150094 tablets in size, shape, color and taste. Both subjects as investigators involved in the trial were blinded to drug allocation. At regular monitoring visits, a study monitor assured that the blind was maintained during study course.

### Statistical Analysis

Based on preclinical [10], [12] and clinical studies [13]–[15] evaluating thyroid hormone action on insulin sensitivity, we estimated to observe a difference of 15 µmol/kg^−1^min^−1^ (∼40%) in peripheral insulin sensitivity (Rd, mean 29 µmol/kg^−1^min^−1^) after TRC150094 treatment. Power calculations indicated that to observe this difference with a two-sided significance level of 0.05 and a power of 80%, we had include at least 10 subjects per study group. Continuous data were analysed with parametric or non-parametric tests depending on the data distribution verified by the Shapiro-Wilk test. Prior to embarking on the efficacy analysis, a separate analysis for trends was performed in both subgroups (10 versus 10 in both groups) to exclude marked heterogeneity of the observed responses between ethnic groups. Within-group comparisons of pre- and post-treatment values were performed using the paired samples Student t-test or Wilcoxon signed ranks test. Between-group comparisons were performed using the unpaired samples t-test or Mann–Whitney U test. Data for qualitative variables are presented as incidence rates (N, number and percent). The data of continuous variables were summarized using measures of central tendency (i.e. mean, median) and dispersion (i.e. standard deviation, range). Statistical analysis was performed using SPSS 19.0 (SPSS, Chicago, IL, USA).

## Results

### Baseline Characteristics

**Figure 1:**
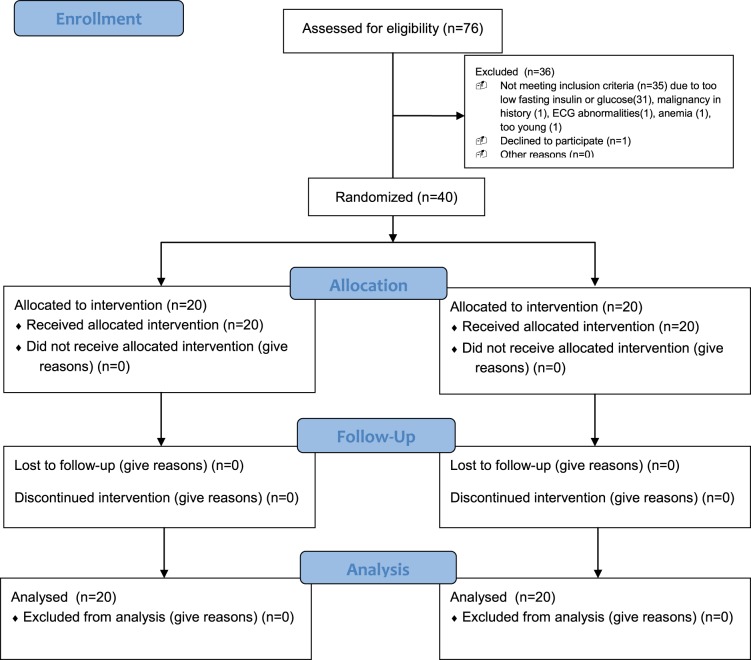
Flowdiagram. Screenfailures in specific; due to malignancy in history, ECG abnormalities at screening, too low fasting insulin or glucose, anemia, age and one withdrawing of consent after screening.

From November 2011 through May 2012 we randomly assigned 40 men to TRC150094 (*n* = 20) or placebo (*n* = 20), all of whom completed the study protocol (Figure 1). At baseline, clinical characteristics were comparable between TRC150094 and placebo group (Table 1). Baseline characteristics were also comparable between Indian and Western European subjects except for BMI, fasting insulin and FFA (Table S1 in File S1).

**Table 1:** Characteristics of Study Subjects at Baseline.

	TRC150094 (N = 20)	Placebo (N = 20)
Age, *y*	49±11	50±10
Weight, *kg*	102±18	103±19
Body mass index, *kg/m^2^*	33.3±4.5	33.6±4.9
Waist, *cm*	112.5±12.0	114.7±11.8
Systolic blood pressure, *mm Hg*	140±8	137±10
Diastolic blood pressure, *mm Hg*	88±4	87±7
Fasting plasma glucose, *mmol/L*	5.6±1.0	5.3±0.6
Fasting plasma insulin, *mU/L*	12±7	14±8
HOMA-IR	2.9±1.8	3.5±2.4
EGP suppr, *%*	60±16	67±15
Rd, *µmol kg* ^−*1*^ *min* ^−*1*^	29±7	28±6
Fasting free fatty acids, *mmol/L*	0.51±0.10	0.52±0.19
Cholesterol, *mmol/L*	4.63±1.03	4.90±0.74
HDLc	0.94±0.24	1.01±0.32
LDLc	2.91±0.82	3.13±0.71
TG	1.60±1.06	1.67±0.72

NOTE. Values are expressed as mean ± standard deviation. No significant differences in clinical variables were found between TRC and Placebo group at baseline, p<0.05. The body mass index is the weight in kilograms divided by the square of the height in meters. HDLc, high-density lipoprotein cholesterol; LDLc, low-density lipoprotein cholesterol; TG, triglycerides.

### Safety Analyses

No serious adverse events were reported and no subjects withdrew from the study after enrolment. The total number of adverse events during the study was similar among the study groups (8 adverse events in both groups). The majority of these events were mild (81%) or moderate (19%). Table S2 in File S1 lists the number, intensity, relationship to treatment and type of adverse events that occurred during the study. No changes in vital signs were observed; blood pressure, heart rate, body temperature and weight remained stable throughout the study (table 2). Liver function tests including ALT, AST and GGT did not change after TRC150094 treatment (table 2). A marginal increase in FT4 in the treatment arm was observed, however, there was no concomitant reduction in TSH (table 2).

**Table 2:** Safety Analyses of Study Subjects at Baseline and After 4 Weeks.

	TRC150094 (N = 20)		Placebo (N = 20)	
	Baseline	Week 4	Baseline	Week 4
Systolic blood pressure, *mm Hg*	140±8	140±9	137±10	133±9
Diastolic blood pressure, *mm Hg*	88±4	88±5	87±7	85±8
Pulse, *beats/min*	76±10	74±9	87±7	75±7
Body temperature, *°C*	36.6±0.5	36.4±0.5	36.6±0.4	36.4±0.4
Body weight, *kg*	101±18	103±21	103±19	102±15
Liver function				
ALT, *U/L*	40±22	39±20	34±10	34±15
AST, *U/L*	29±12	27±11	29±13	26±9
GGT, *IU/L*	44±31	43±28	46±31	47±34
Thyroid function				
FT3, *pmol/L*	4.27±1.02	4.88±1.29	4.45±0.68	5.11±1.03
FT4, *pmol/L*	11.70±2.12	12.57±1.96*	12.1±1.84	12.3±1.94
TSH, *mIU/L*	2.10±0.00	2.23±1.14	1.86±0.67	2.01±1.06

NOTE. Values are expressed as mean ± standard deviation.

*Nonparametric test showed a significant increase in FT4 after TRC150094 (p = 0.025). ALT, alanine aminotransferase; AST, aspartate aminotransferase; GGT, gamma glutamyltransferase; FT3, free trio-iodothyronine; FT4, free thyroxine; TSH, thyroid stimulating hormone (thyrotropin).

### Efficacy Analyses in Subjects at Increased Cardiometabolic Risk

#### Effect of TRC150094 on insulin sensitivity

**Figure 2:**
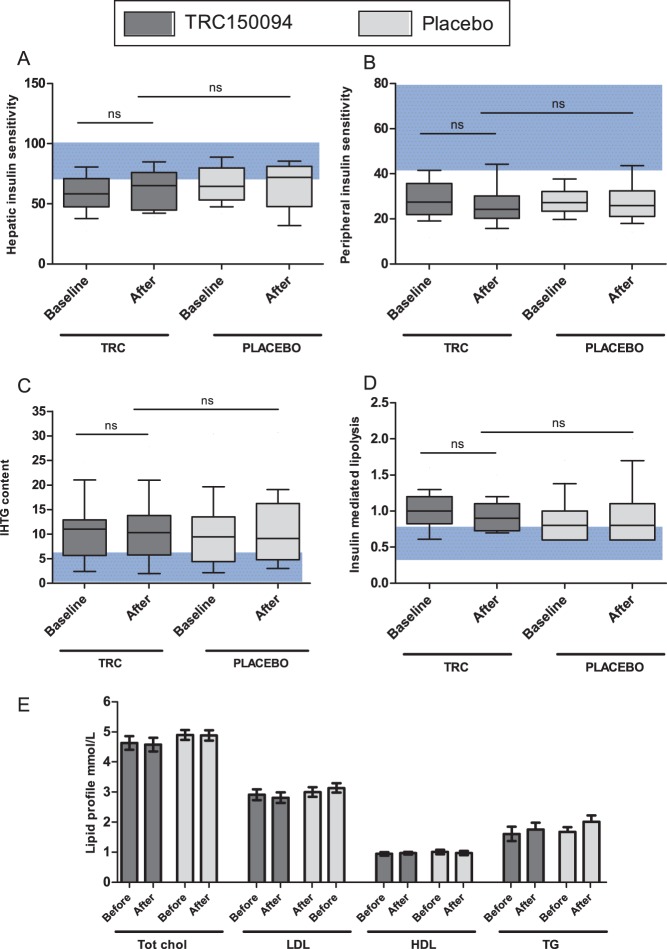
Efficacy data TRC150094 in males with increased cardiometabolic risk. **A–D;** Box plots of hepatic insulin sensitivity (suppression of EGP %), peripheral insulin sensitivity (Rd umol*kg^−1^min^−1^), hepatic fat content (IHTG %) and insulin mediated suppression of lipolysis (suppression of lipolysis %) before after TRC administration. Blue background depicts reference values in healthy population, based on historical data [16]–[19]. **E**; Bar graph of lipid profile showing no improvement after TRC150094 administration.

At baseline, male subjects were characterized by markedly impaired hepatic and peripheral insulin sensitivity, compared to reference values observed in healthy, non-obese control subjects [16]–[19] (Figure 2 A–B). Hepatic insulin sensitivity was expressed as the suppression of Endogenous Glucose Production (EGP). After TRC150094 administration there was no improvement in suppression of endogenous glucose production (mean EGP suppression from 59.5 to 62.1%; p = 0.477) (Figure 2A), whereas peripheral insulin sensitivity (expressed as the rate of glucose disappearance (Rd)) was not altered upon TRC150094 administration (mean Rd from 28.8 to 26.4 µmol/kg^−1^min^−1^, p = 0.185) (Figure 2B). Although T2’s mechanism of action is expected to stimulate lipolysis and FAO, TRC150094 administration did not result in differences in fasting plasma FFA (mean FFA from 0.51 to 0.51 mmol/L, p = 0.887) or in insulin-mediated suppression of lipolysis (lipolysis suppression from 57 to 54%, p = 0.102) (Figure 2C). Overview of efficacy results in glucose kinetics, lipolysis and glucoregulatory hormones at baseline and after TRC administration are provided in Table S3 in File S1. Additional efficacy analysis for Indian and Western European subjects separately demonstrated comparable results (data not shown).

#### Effect of TRC150094 on IHTG content and lipid profile

Intrahepatic triglyceride (IHTG) content was measured with ^1^H MRS. At baseline, mean IHTG content was 10.6% (±6.4%) in the whole group. After 4-weeks of treatment, IHTG was unaltered in both the TRC150094 and the placebo group (Figure 2). Similarly, no change in lipid profile, i.e. total cholesterol, LDLc, HDLc or TG, was detected in the patients on either TRC150094 or placebo (Figure 2). Responses divided per treatment arm are provided in Table S4 in File S1.

### Subgroup Analysis in Subjects with Severe Metabolic Derangement

To evaluate whether the response differed between subjects with mild and severe metabolic derangement, we analyzed the subjects with a mean TG above 1.64 mmol/L compared to those below the mean plasma TG. Subgroup analysis showed a numerical reduction of IHTG content in the highest TG group (absolute IHTG from 12.7% (±3.9) to 11.8% (±4.3) (p = 0.378) with a relative IHTG change of –6.3% (p = 0.682), which did not reach statistical significance. In the subjects below mean TG, IHTG levels remained stable (absolute IHTG from 9.9% (±6.9) to 10.4% (±8.1), as reflected by a relative IHTG change of +3.6% (p = 0.759). Changes in hepatic and peripheral insulin sensitivity were not significantly different between upper versus lower TG groups. Finally, TG levels decreased in the upper TG group following TRC150094 (from 2.66±1.17 to 2.26±1.31 mmol/L, p = 0.012) whereas no change was observed in the lower TG group. See Table S5 in File S1 for an overview of the subgroup analyses.

## Discussion

In the present study we show that short-term administration of TRC150094 dosed 50 mg once daily is safe and well tolerated. Neither hepatic, nor peripheral insulin sensitivity improved in subjects at an increased cardiometabolic risk. In line, IHTG content and plasma lipid profiles were unaltered following 4 weeks of TRC150094 administration. A subgroup analysis in subjects with TG levels above the mean did reveal a significant reduction in TG levels but no changes in insulin sensitivity or IHTG. Collectively, these data show that, in contrast to the potent metabolic effects in experimental models, TRC150094 at a dose of 50 mg daily does not improve the metabolic homeostasis in subjects at an increased cardiometabolic risk. Further studies are needed to evaluate whether TRC150094 may have an effect in subjects with more severe metabolic derangement, such as overt diabetes mellitus and hypertriglyceridemia.

Following 4 weeks of TRC150094 administration at a dose of 50 mg once daily, neither hepatic nor peripheral insulin sensitivity changed. In line, hepatic fat content and lipid profile were unaltered. This apparent discrepancy between the marked impact of TRC150094 on glycemic profile, hepatic fat accumulation and serum lipids in experimental protocols [8], [9], [20], [21] and the absence of any metabolic improvement in the present clinical study may have several explanations, consisting of the mechanism of action, the dose and concentration of TRC150094 and study population.

First, the mechanism of action of di-iodothyronine (T2). The biologically active thyroid hormone tri-iodothyronine (T3) exerts its effects via specific nuclear receptors; namely TR α and β. [2] T2 has a 50–400 times lower affinity for TR than T3, making it unlikely that TR activation contributes to the effects of T2. [22] Extensive preclinical work, however, did substantiate a rapid effect of T2 on energy expenditure in rats, which were shown to be mediated by direct effects on mitochondria independent of classical nuclear thyroid receptors. [23] The T2 mimetic TRC150094, which is also associated with minimal TR transcriptional activation, [8] was observed to increase whole body mitochondrial fatty acid oxidation (FAO) and resting metabolic rate (RMR) in rats, leading to marked improvements of glucose and lipid homeostasis. [8], [9], [20], [21] In contrast, data on the (patho)physiological relevance of T2 in humans are absent. [24] In fact, proof for a receptor-independent effect of T2 in humans is lacking altogether. In the present study, TRC150094 also failed to increase FAO in terms of decreased plasma FFA and less insulin mediated suppression of lipolysis. As this study was of shorter duration, i.e. 28 days, we can not exclude the possibility that the receptor-independent activities of T2 may become apparent in humans after a longer duration. [22].

Secondly, in the current study 50 mg once daily dosed was selected based on drug exposure (AUC) as well as safety and tolerability data obtained from earlier animal models [8]–[10] and a phase I Multiple Ascending Dose study in humans (data not published). In previous animal studies, the plasma exposure in which significant effects on insulin sensitivity and hepatic lipid content were observed ranged between 3.8 to 11.8 µg*h/ml. Steady state exposure of TRC150094 in humans was observed between 2.7 to 8.0 µg*h/ml after administration of 50 mg once a day for 28 days. Nevertheless, the selected dose of TRC150094 50 mg once daily may have been insufficient. This could be explained by human equivalent dose (HED) calculation [25] from animal efficacy studies. The current dose of 0.5 mg/kg (mean weight approx 100 kg) in humans may be at the lower end, since conversion for drug dosage between rats and humans indicates a HED of approximately 4 mg/kg. [25] Thus efficacy of TRC150094 at a higher dose of 75 to 100 mg once daily needs further exploration in future studies.

Thirdly, we have investigated treatment effects in patients with insulin resistance indicated by a reduced EGP suppression as well as peripheral glucose disposal rate. Previous clamp studies have shown that these patients have the potency towards improving their metabolic homeostasis in only short duration interventions in even smaller cohorts [26]. We therefore do not assume that present sample size was insufficient to observe a favorable effect. Also, inhomogeneity between Indian and Western Europeans is not a likely explanation for a lack of effect in this study, since separate analysis of these groups did not indicate otherwise. However, a subgroup analyses in subjects with severe metabolic derangement, [27] identified via plasma TG >1.64 mmol/l at baseline, did reveal a positive trend after TRC150094 treatment. Whereas the high-TG subjects showed comparable hepatic fat content levels at baseline compared to the low TG subjects, hepatic fat content following TRC150094 administration was reduced numerically by 6% in the high TG subjects versus no change in the subjects with lower TG levels. In line, hepatic and peripheral insulin sensitivity did show a trend towards improvement (respectively +2.69% and +3.61%). Thus, it cannot be ruled out that an effect may have been observed in case of selection of metabolic syndrome patients with markedly elevated TG levels.

Overall, TRC150094 administration was well tolerated and showed no safety concerns. The modest changes in FT4 were unexpected [28], since the affinity of TRC150094 for TR α and β is extremely low. Most importantly, we can exclude any biological relevance since no decrease in TSH was observed. Besides, other clinical manifestations of TR activation were absent, such as changes in blood pressure, heart rate, body temperature and body weight following TRC150094 administration.

In conclusion, in the present phase 2, randomized double-blind controlled trial we show that TRC150094 did not improve insulin sensitivity and lipid metabolism or decrease hepatic steatosis in obese insulin resistant subjects with an increased cardiometabolic risk. Since subgroup analysis in subjects with high triglyceride levels provided a trend towards improvement, future studies should address the potential impact of TRC150094 administration at higher dose, particularly in patients at a high cardiometabolic risk with elevated TG levels.

## Supporting Information

File S1 **Methods S1**, Hyperinsulinemic euglycemic clamp, Glucose and glucoregulatory hormones measurements, and ^1^H-MRS.**Table S1, Characteristics of study subjects at baseline between ethnicities. Table S2, Number of patients with adverse events. Table S3, Glucose kinetics, glucoregulatory hormones in TRC and placebo group at baseline and after 4 weeks treatment. Table S4, IHTG content and lipid profiles in TRC and placebo group at baseline and week 4. Table S5, Subgroup analyses in subjects with high triglycerides.**
(DOC)

Checklist S1 **CONSORT checklist.**
(DOC)

Protocol S1 (PDF)
