# Supplementary material for: The Effect of a Diiodothyronine Mimetic on Insulin Sensitivity in Male Cardiometabolic Patients: A Double-Blind Randomized Controlled Trial
Source: PLoS One. 2014 Feb 21;9(2):e86890. doi: 10.1371/journal.pone.0086890 (PMC3931609; doi:10.1371/journal.pone.0086890)
Supplement: File S1 — Methods S1, Hyperinsulinemic euglycemic clamp, Glucose and glucoregulatory hormones measurements, and 1H-MRS.Table S1, Characteristics of study subjects at baseline between ethnicities. Table S2, Number of patients with adverse events. Table S3, Glucose kinetics, glucoregulatory hormones in TRC and placebo group at baseline and after 4 weeks treatment. Table S4, IHTG content and lipid profiles in TRC and placebo group at baseline and week 4. Table S5, Subgroup analyses in subjects with high triglycerides. (DOC) [file pone.0086890.s001.doc]

**Supporting Information file S1**

**Supporting Methods**

*Hyperinsulinemic euglycemic clamp*

Prior to the study day, all subjects refrained form vigorous exercise for 48 hours. After an overnight fast, subjects were admitted to the metabolic ward of the study centre at 07:15 am. A catheter was inserted in an antecubital vein for infusion of stable isotope tracers, insulin and glucose. Another catheter was inserted into a contralateral hand vein and kept in a thermoregulated (60°C) Plexiglas box for sampling of arterialised venous blood. Saline was infused as NaCl 0.9% at a rate of 50 ml/h to sustain catheter patency. [6,6-²H_2_]glucose and [1,1,2,3,3-^2^H_5_]glycerol were infused as tracers (>99% enriched; Cambridge Isotopes, Andover, MA, USA) to study glucose kinetics and lipolysis (total triacylglycerol hydrolysis), respectively. At time 0 (08:30 am) blood samples were drawn for determination of background enrichments, where after a continuous infusion of isotopes was started ([6,6-²H_2_]glucose and [1,1,2,3,3-^2^H_5_]glycerol, both at a rate of 0.11 μmol*kg^−1^ min^−1^, with a priming dose equivalent to 80 min of infusion) and continued until the end of study. After an equilibration time of 150 min, three blood samples were taken for the measurement of isotope enrichments and one for the measurement of glucoregulatory hormones and NEFA. Thereafter, a two-step hyperinsulinaemic–euglycaemic clamp was started. A continuous infusion of insulin (Actrapid 100 U/ml; Novo Nordisk Farma, Alphen aan de Rijn, the Netherlands) was started for 130 min at the rate of 20 mU [m^2^ body surface area]^−1^min^–1^, followed by an infusion of insulin at a rate of 60 mU [m^2^ body surface area]^−1^min^–1^ for another 130 min. Plasma glucose levels were measured every 10 min at the bedside. Glucose was infused as 20% glucose at a variable rate, to maintain a plasma glucose concentration of 5.0 mmol/l. [6,6-²H_2_]glucose was added to the 20% glucose solution to achieve glucose enrichments of 1% to approximate the values for enrichment reached in plasma and thereby minimise changes in isotopic enrichment due to changes in the infusion rate of exogenous glucose.[1] During the last 40 min of both hyperinsulinaemic periods, blood samples were drawn at 5 min intervals for determination of isotope enrichments and glucoregulatory hormones. During the study day, all subjects remained fasted but were allowed to drink water.

*Glucose and glucoregulatory hormones measurements*

Plasma glucose concentrations were measured with the glucose oxidase method using a YSI analyzer. [6,6-^2^H_2_]glucose enrichment (tracer-to-tracee ratio) was measured as reported earlier[2] with an intra-assay variation of 0.5–1% and an inter-assay variation of 1% and a detection limit of 0.04%. [1,1,2,3,3-^2^H_5_]glycerol enrichment was determined with an intra-assay variation of 1–3% for glycerol and 4% for [1,1,2,3,3-^2^H_5_]glycerol, and inter-assay variation of 2–3% for glycerol and 7% for [1,1,2,3,3-^2^H_5_]glycerol, as reported earlier.[3] Insulin was determined on an Immulite 2000 system (Diagnostic Products, Los Angeles, CA, USA). Insulin was measured with a chemiluminescent immunometric assay with intra-assay variation of 3–6%, inter-assay variation of 4–6% and detection limit of 15 pmol/l. Calculations and statistics HOMA of insulin resistance (HOMA-IR) was calculated using the formula described previously by Matthews et al.[4] Endogenous glucose production (EGP) and peripheral glucose uptake (rate of disappearance [Rd]) were calculated using the modified forms of the Steele equations.[5] EGP and Rd were expressed as μmol kg^−1^min^−1^. Insulin clearance was calculated as the rate of insulin infusion (mU [m^2^ body surface area]^−1^ min^−1^) divided by the mean plasma insulin concentration during the clamp.[6] Lipolysis (glycerol turnover) was calculated using formulas for steady-state kinetics adapted for stable isotopes and was expressed as μmol kg^−1^min^−1^.[3,5] Lipolysis was assessed as percentage change in rate of appearance of glycerol from the basal to low dose insulin-stimulated state. Plasma FFA concentrations were measured with an enzymatic colorimetric method (NEFA-C test kit; Wako Chemicals GmbH, Neuss, Germany) (intra-assay variation 1%, total-assay variation 4-15%; detection limit 0.02 mmol/L)

*^1^H MRS*

¹H-MRS spectra were acquired using a 3.0 T Intera (Philips, Best, the Netherlands). During the measurements, subjects remained in the supine position within the MRI scanner. IHTG content was obtained using single-voxel ¹H-MRS, using a body array coil as the transmitter and phased surface coils as receivers. MRS measurements were acquired during breathhold, using single-voxel stimulated acquisition mode (TE/TR 20/3.000 ms, six acquisitions). Volumes of interest in the liver were located away from major vascular structures and bile ducts. Voxel size was 27 mm^3^. The water and fat resonance peaks, located at 4.65 and 1.3 ppm, were integrated using jMRUI software[7], and relative fat content was expressed as the ratio of the fat peak area over the cumulative water and fat peak areas. Calculated peak areas of water and fat were corrected for T2 relaxation (T2water, 34 ms; T2fat, 68 ms[8]) and the percentage hepatic fat content was calculated.[9]

**Supporting Tables**

| **Table S1 –** Characteristics of Study Subjects at Baseline Between Ethnicities | | | | |
| --- | --- | --- | --- | --- |
|  | Indian ( N = 20) | | Caucasian ( N = 20) | |
|  | TRC150094  ( N = 10 ) | Placebo  ( N = 10 ) | TRC150094  ( N = 10 ) | Placebo  ( N = 10 ) |
| Age, *y* | 41 ± 7 | 43 ± 6 | 57 ± 6 | 57 ± 8 |
| Weight, *kg* | 87 ± 7 | 90 ± 11 | 116 ± 13 | 116 ± 16 |
| Body mass index, *kg/m^2^** | 30.2 ± 2.7 | 31.1 ± 3.3 | 36.4 ± 3.7 | 36.0 ± 5.1 |
| Waist, *cm** | 105 ± 6 | 107 ± 7 | 120 ± 11 | 123 ± 11 |
| Fasting plasma glucose, *mmol/L* | 5.7 ± 1.3 | 5.3 ± 0.7 | 5.5 ± 0.7 | 5.3 ± 0.4 |
| Fasting plasma insulin, *mU/L ** | 8 ± 3 | 13 ± 9 | 17 ± 8 | 16 ± 5 |
| HOMA-IR | 2.0 ± 1.0 | 3.3 ± 2.8 | 3.7 ± 2.1 | 3.7 ± 2.0 |
| Cholesterol, *mmol/L* | 4.68 ± 1.09 | 4.93 ± 0.90 | 4.59 ± 1.02 | 4.87 ± 0.59 |
| HDLc | 1.02 ± 0.30 | 1.04 ± 0.42 | 0.87 ± 0.14 | 0.98 ± 0.21 |
| LDLc | 2.89 ± 0.81 | 3.03 ± 0.87 | 2.93 ± 0.87 | 3.24 ± 0.52 |
| TG | 1.50 ± 1.16 | 1.91 ± 0.91 | 1.72 ± 1.00 | 1.44 ± 0.39 |
| Plasma free fatty acids, *mmol/L** | 0.52 ± 0.12 | 0.40 ± 0.09 | 0.50 ± 0.09 | 0.65 ± 0.18 |
| Systolic blood pressure, *mm Hg* | 139 ± 2 | 139 ± 2 | 145 ± 10 | 140 ± 11 |
| Diastolic blood pressure, *mm Hg* | 89 ± 1 | 89 ± 1 | 90 ± 4 | 89 ± 6 |

NOTE. Values are expressed as mean ± standard deviation.

*Baseline characteristics were comparable between Indian and Caucasian subjects except for BMI, waist, fasting insulin and FFA. The body mass index is the weight in kilograms divided by the square of the height in meters. HDLc, high-density lipoprotein cholesterol; LDLc, low-density lipoprotein cholesterol; TG, triglycerides.

**Table S2 –** Number of patients with adverse events

|  | TRC150094 ( N = 20) | Placebo ( N = 20) |
| --- | --- | --- |
| Serious adverse event | 0 | 0 |
| Adverse event | 8 | 8 |
| Intensity |  |  |
| Mild | 6 | 7 |
| Moderate | 2 | 1 |
| Relationship to TRC150094/Placebo |  |  |
| Not likely | 4 | 3 |
| Possible | 4 | 5 |
| Probable | 0 | 0 |
| Event |  |  |
| Back pain | 2 | 2 |
| Blurred vision | 1 | 1 |
| Diarrhea | 1 | 1 |
| Dry mouth | 0 | 1 |
| Fatigue | 1 | 0 |
| Flu like symptoms | 0 | 2 |
| Headache | 1 | 2 |
| Heartburn | 1 | 0 |
| Hip pain | 0 | 2 |
| Increased appetite | 1 | 0 |
| Insomnia | 0 | 1 |
| Polyuria | 1 | 0 |
| Rash | 1 | 0 |

**Table S3 –** Glucose Kinetics, Glucoregulatory hormones in TRC and Placebo Group at Baseline and After 4 Weeks Treatment

|  | TRC ( N = 20) | | Placebo ( N = 20 ) | |
| --- | --- | --- | --- | --- |
|  | Baseline | 4 weeks | Baseline | 4 weeks |
| Glucose, *mmol/L* |  |  |  |  |
| Basal | 5.2 (4.1-8.4) | 5.5 (4.5-8.9) | 5.1 (4.4-6.5) | 5.3 (4.6-7.8) |
| Step 1 | 5.1 (4.7-6.4) | 5.1 (4.8-5.8) | 5.0 (4.8-5.6) | 5.1 (4.9-6.2) |
| Step 2 | 5.0 (4.8-5.5) | 5.1 (4.6-5.4) | 4.9 (4.4-5.4) | 5.0 (4.6-5.8) |
| Insulin, *mU/L* |  |  |  |  |
| Basal | 11 (2-28) | 11 (4-29) | 13 (4-37) | 13 (2-24) |
| Step 1 | 32 (19-68) | 30 (17-81) | 39 (19-149) | 40 (17-55) |
| Step 2 | 96 (62-225) | 97 (54-197) | 98 (58-145) | 106 (51-162) |
| EGP, *μmol kg^-1^min^-1^* |  |  |  |  |
| Basal | 9.1 (7.5-13.4) | 9.0 (7.4-13.4) | 8.7 (7.3-13.1) | 8.7 (6.8-11.9) |
| Step 1 | 3.9 (1.2-3.8) | 2.7 (0.6-7.7) | 3.2 (0.7-5.7) | 2.4 (0-9.3) |
| EGP suppr, *%* | 60 (52-67) | 62 (55- 70) | 67 (60-74) | 61 (50-72) |
| Rd, *μmol kg^-1^min^-1^* | 27.5 (11.7-48.0) | 24.2 (11.1-46.9) | 27.3 (16.6-39.4) | 29.9 (14.2-45.5) |
| Plasma FFA, *mmol/L* | 0.51 (0.36-0.77) | 0.51 (0.26-0.67) | 0.52 (0.25-0.94) | 0.47 (0.21-0.69) |
| Lipolysis, *μmol kg^-1^min^-1^* |  |  |  |  |
| Basal | 2.1 (1.2-3.2) | 2.2 (1.3-4.6) | 2.2 (1.1-3.3) | 2.2 (1.4-4.1) |
| Step 1 | 0.9 (0.6-1.7) | 1.0 (0.6-3.2) | 1.0 (0.6–1.6) | 0.9 (0.5-1.5) |
| Lipolysis suppr, *%* | 57 (32-77) | 54 (20-77) | 53 (14-72) | 56 (33-78) |

NOTE. Values are expressed medians (minimum – maximum). Step 1 is measurements during low dose insulin infusion and step 2 is measurements during high dose insulin infusion during hyperinsulinemic euglycemic clamp.

**Table S4 –** IHTG content and lipid profiles in TRC and Placebo Group at Baseline and Week 4

|  | TRC ( N = 20) | | Placebo ( N = 20 ) | |
| --- | --- | --- | --- | --- |
|  | Baseline | 4 weeks | Baseline | 4 weeks |
| **Liver** |  |  |  |  |
| IHTG content, % | 10.8 ± 6.1 | 10.9 ± 6.9 | 10.4 ± 6.9 | 10.6 ± 7.2 |
| **Serum** |  |  |  |  |
| Tot al cholesterol, *mmol/L* | 4.63 ± 1.03 | 4.58 ± 1.02 | 4.90 ± 0.74 | 4.88 ± 0.79 |
| LDLc | 2.91 ± 0.82 | 2.81 ± 0.79 | 3.13 ± 0.71 | 3.00 ± 0.71 |
| HDLc | 0.94 ± 0.24 | 0.97 ± 0.21 | 1.01 ± 0.32 | 0.97 ±0.31 |
| TG | 1.61 ± 1.06 | 1.75 ± 1.00 | 1.67 ± 0.72 | 2.01 ± 0.94 |

NOTE. Values are expressed mean ± standard deviation.

**Table S5** – Subgroup analyses in Subjects with High TG

|  | TRC ( N = 7) | | | Placebo ( N = 8 ) | | |
| --- | --- | --- | --- | --- | --- | --- |
|  | Baseline | 4 weeks | Rel  change | Baseline | 4 weeks | Rel  change |
| EGP suppr, *%* | 56.2  ± 10.5 | 58.3  ± 15.4 | + 2.69 | 70.0  ± 17.2 | 64.4  ± 17.9 | - 6.73 |
| Rd, *μmol kg^-1^min^-1^* | 25.9  ± 8.1 | 27.0  ± 11.4 | + 3.61 | 26.5  ± 5.9 | 25.5  ± 5.4 | - 0.44 |
| IHTG, *%* | 12.7  ± 3.9 | 11.8  ± 4.3 | - 6.31 | 9.3  ± 4.0 | 9.5  ± 5.4 | - 2.42 |
| Total cholesterol, *mmol/L* | 4.87  ± 1.33 | 4.60  ± 1.52 | - 6.46 | 5.13  ± 0.98 | 5.23  ± 0.76 | + 2.93 |
| LDLc | 2.79  ± 1.08 | 2.67  ± 1.14 | - 4.60 | 3.25  ± 0.81 | 3.29  ± 0.69 | - 0.22 |
| HDLc | 0.86  ± 0.12 | 0.90  ± 0.11 | + 5.89 | 0.82  ± 0.13 | 0.83  ± 0.19 | + 2.44 |
| TG | 2.66  ± 1.17 | 2.26  ± 1.31 | - 18.6 | 2.33  ± 0.66 | 2.47  ± 0.61 | + 7.23 |

Subgroup analyses of subjects with serum TG >1.64 mmol/L at baseline.

NOTE. Values are expressed mean ± standard deviation. Relative change is the mean relative change in percentage.

Reference List

1. Finegood DT, Bergman RN, Vranic M (1987) Estimation of endogenous glucose production during hyperinsulinemic-euglycemic glucose clamps. Comparison of unlabeled and labeled exogenous glucose infusates. Diabetes 36: 914-924.

2. Ackermans MT, Pereira Arias AM, Bisschop PH, Endert E, Sauerwein HP et al. (2001) The quantification of gluconeogenesis in healthy men by (2)H2O and [2-(13)C]glycerol yields different results: rates of gluconeogenesis in healthy men measured with (2)H2O are higher than those measured with [2-(13)C]glycerol. J Clin Endocrinol Metab 86: 2220-2226.

3. Ackermans MT, Ruiter AF, Endert E (1998) Determination of glycerol concentrations and glycerol isotopic enrichments in human plasma by gas chromatography/mass spectrometry. Anal Biochem 258: 80-86. S0003-2697(97)92535-5 [pii];10.1006/abio.1997.2535 [doi].

4. Matthews DR, Hosker JP, Rudenski AS, Naylor BA, Treacher DF et al. (1985) Homeostasis model assessment: insulin resistance and beta-cell function from fasting plasma glucose and insulin concentrations in man. Diabetologia 28: 412-419.

5. Steele R (1959) Influences of glucose loading and of injected insulin on hepatic glucose output. Ann N Y Acad Sci 82: 420-430.

6. Kotronen A, Vehkavaara S, Seppala-Lindroos A, Bergholm R, Yki-Jarvinen H (2007) Effect of liver fat on insulin clearance. Am J Physiol Endocrinol Metab 293: E1709-E1715. 00444.2007 [pii];10.1152/ajpendo.00444.2007 [doi].

7. Naressi A, Couturier C, Devos JM, Janssen M, Mangeat C, et al. (2001) Java-based graphical user interface for the MRUI quantitation package. MAGMA 12: 141-152. S1352866101001119 [pii].

8. de Bazelaire CM, Duhamel GD, Rofsky NM, Alsop DC (2004) MR imaging relaxation times of abdominal and pelvic tissues measured in vivo at 3.0 T: preliminary results. Radiology 230: 652-659. 10.1148/radiol.2303021331 [doi];230/3/652 [pii].

9. Szczepaniak LS, Babcock EE, Schick F, Dobbins RL, Garg A, Burns DK, McGarry JD, Stein DT (1999) Measurement of intracellular triglyceride stores by H spectroscopy: validation in vivo. Am J Physiol 276: E977-E989.
