# Supplementary material for: The Effect of a Diiodothyronine Mimetic on Insulin Sensitivity in Male Cardiometabolic Patients: A Double-Blind Randomized Controlled Trial
Source: PLoS One. 2014 Feb 21;9(2):e86890. doi: 10.1371/journal.pone.0086890 (PMC3931609; doi:10.1371/journal.pone.0086890)
Supplement: Protocol S1 — (PDF) [file pone.0086890.s003.pdf]

Hyperinsulinemic Euglycemic Clamp Protocol

Title

A Phase 2A, Double-blind, Placebo-controlled, Randomized Study to Evaluate the Safety and Efficacy of TRC150094 in Increasing Insulin Sensitivity in Male patients with increased Cardiometabolic Risk

**Study Number:**	CT/P015/CMR/2010/02_01
**Protocol Version:**	3.0
**Protocol Date:**	18/11/2011
**CTRI Number:**	REFCTRI-2010 002973
**Investigational Medicinal Product:**	TRC150094
**Indication:**	Cardiometabolic Risk associated with non-traditional risk factors
**Clinical Phase:**	Phase 2A
**Sponsor:**	Torrent Pharmaceuticals Limited<br>Torrent Research Centre<br>Village Bhat<br>Dist Gandhinagar<br>Gujarat, India<br>Tel: +91 79 23969100<br>Fax: +91 79 23969135
**Principal Investigators:**	Prof. E.S.G Stroes (AMC, The Netherlands)<br>Dr. Dharmesh Domadia (Veeda CR, India)

The information contained herein is strictly confidential and must not be disclosed, copied, submitted for publication, or used for any purpose without the sponsor's prior written authorisation.

INVESTIGATOR SIGNATURE PAGE

A Phase 2A, Double-blind, Placebo-controlled, Randomized Study to Evaluate the Safety and Efficacy of TRC150094 in Increasing Insulin Sensitivity in Male patients with increased Cardiometabolic Risk

I confirm that I have read and I understand this protocol and other appropriate related documentation, including the Investigator's Brochure for TRC150094. I agree that the available pre-clinical information on the investigational product is adequate to support the proposed clinical trial. I agree to perform this study in accordance with this protocol, the ethical principles that have their origin in the Declaration of Helsinki, the International Conference on Harmonisation (ICH) guideline on Good Clinical Practice (GCP), and the applicable regulatory requirement(s). I will also appropriately direct and assist the personnel at the trial site who will be involved in the conduct of the study.

Prof. E.S.G Stroes Date

Department of Vascular Medicine, AMC

Amsterdam, The Netherlands

SPONSOR APPROVAL SIGNATURE PAGE

A Phase 2A, Double-blind, Placebo-controlled, Randomized Study to Evaluate the Safety and Efficacy of TRC150094 in Increasing Insulin Sensitivity in Male patients with increased Cardiometabolic Risk

Approved by:

______________________________ ______________

Dr. Chaitanya Dutt Date

Director, R&D

Torrent Pharmaceuticals Limited
Torrent Research Centre
Village Bhat
Dist Gandhinagar 382 428
Gujarat
India

SPONSOR CONTACT DETAILS*

Study Monitor: **Dr. Lalit Lakhwani**

Scientist I, Clinical Research

Tel: +91 79 23969100 Ext. 194

Mobile: +91 9227400443

Email: lalitlakhwani@torrentpharma.com

Fax: +91 79 23969135

Project Manager: **Mr. Kashyap Avashia**

AGM, TCM-Discovery

Tel: +91 79 23969100 Ext. 566

Mobile: +91 9727704562

Email: kashyapavashia@torrentpharma.com

Fax: +91 79 23969135

Medical Experts:

Prof. E.S.G. Stroes MD, PhD

Department of Vascular Medicine, AMC

Tel: +31 20 566 5978

Email- E.S.G.Stroes@amc.uva.nl

Dr. Chaitanya Dutt

Director, R&D

Tel: +91 79 23969100 Ext. 101

Mobile: +91 9825606901

Email: cdutt@torrentpharma.com

Fax: +91 79 23969135

24-Hour Emergency

Contacts/ SAE Reporting:

The 24 hour Emergency Contacts will be contacted as per the order mentioned below:

Dr. Lalit Lakhwani

Scientist I, Clinical Research

Tel: +91 79 23969100 Ext. 194

Mobile: +91 9227400443

Email: lalitlakhwani@torrentpharma.com

Fax: +91 79 23969135

Dr. KumarPrafull Chandra

AGM, Clinical Research

Tel: +91 79 23969100 Ext. 193

Mobile: +91 9909004214

Email: kumarprafullchandra@torrentpharma.com

Fax: +91 79 23969135

Dr. Ambrish Srivastava

General Manager, Clinical Research

Tel: +91 79 23969100 Ext. 181

Mobile: +91 9879107948

Email: ambrishsrivastava@torrentpharma.com

Fax: +91 79 23969135

* contact address for all above:

Torrent Research Centre
Village Bhat, Dist Gandhinagar 382 428
Gujarat, INDIA

CONTACT DETAILS FOR PERSONNEL AT STUDY SITES

Academic Medical Centre, Amsterdam

Role	Name	Telephone	Email
Principal Investigator	**Prof. E.S.G. Stroes MD, PhD**<br>**Department of Vascular Medicine, AMC**	**+31 20 566 5978**	**E.S.G.Stroes@amc.uva.nl**
Independent physician	**M.D.trip MD, PhD**<br>**Department of Vascular Medicine AMC**	**+31 20 566 65882**	**M.D.Trip@amc.uva.nl**
Project Manager	**H. Suwier**<br>**ClinResearch Consulting BV.BVBA**	**0032-50673524**<br>**Mobile +32475687662**	**Heidisuwier.jpl@gmail.com**

Veeda Clinical Research, Ahmedabad

Role	Name	Telephone	Email
Principal Investigator	**Dr. Dharmesh Domadia**	**079-30013060**<br>**Mobile: +91 9879590828**	**Dharmesh.domadia@veedacr.com**
Co-investigator	**Dr. Hardik Dave**	**079-30013010 (Ext 245)**	**Hardik.dave@veedacr.com**
Project Manager	**Mr. Prakash Patel**	**079-30013010 (Ext 244)**<br>**Mobile:+91 9909930321**	**Prakash.patel@veedacr.com**

DETAILS OF STUDY FACILITIES AND PERSONNEL

A. ACADEMIC MEDICAL CENTRE, AMSTERDAM

**Principal investigator**	E.S.G. Stroes MD, PhD<br>Department of Vascular Medicine, AMC<br>Meibergdreef 9, room F4-275<br>1100 DD Amsterdam, The Netherlands<br>E-mail:E.S.G.Stroes@amc.uva.nl<br>Tel: +31 20 566 5978, pager nr: 8158287
**Independent physician**	M.D.trip MD, PhD<br>Department of Vascular Medicine AMC<br>Meibergdreef 9, room F4-109<br>1100 DD Amsterdam, The Netherlands<br>E-mail: M.D.Trip@amc.uva.nl<br>Tel: +31 20 566 65882, pager nr: 8159520
**Laboratory sites**	W. Schornagel<br>LAKC, AMC<br>Meibergdreef 9, room B1-238<br>1100 DD Amsterdam, The Netherlands<br>E-mail: w.schornagel@amc.uva.nl<br>Tel: +31 20 5665866<br>M.T.Ackermans<br>Lab. Spec. Endocrinology, AMC<br>Meibergdreef 9, room F2-131.3<br>1100 DD Amsterdam, The Netherlands<br>E-mail: M.T.Ackermans@amc.uva.nl<br>Tel: +31 20 5665924 pager nr: 8165924<br>G.M.Dallinga-thie<br>Lab. Exp. Vascular medicine<br>Meibergdreef 9, room K1-262<br>1100 DD Amsterdam, The Netherlands<br>E-mail: G.M.Dallinga@amc.uva.nl<br>Tel: +31 20 5665158
**Pharmacy**	E.M.Kemper, PhD<br>Pharmacy, AMC<br>Meibergdreef 9, room E0B-100<br>1100 DD Amsterdam, The Netherlands<br>E-mail: E.M.Kemper@amc.uva.nl<br>Tel: +31 20 5667955

B. VEEDA CLINICAL RESEARCH

**Principal Investigator**	Dr. Dharmesh Domadia
	Veeda Clinical Research, Ahmedabad
**Co-investigator**	Dr. Hardik Dave
**Project manager**	Mr. Prakash Patel
**Pharmacy**	Mr. Pankaj Sojitra
**Clinical Laboratory Tests (Safety)**	Supratech Micropath Laboratory & Research Institute<br>'KEDAR' Opp. Krupa Petrol Pump<br>Nr. Parimal Garden<br>Ahmedabad – 380 006, India.<br>Phone No.: +91-79-2640 8181<br>+91-79-2640 8182<br>Fax No.: +91-79-2640 9292
**Radiological Tests**	Shachi Digital X-Ray, Sonography and Color Doppler Clinic<br>F-2, 3, Balaji centre, Opp. Gurukul, Drive-in road,<br>Memnagar, Ahmedabad-380 052, India.<br>Phone: +91-79-2749 1622
**Emergency Care Hospital**	Sterling Hospital<br>Memnagar<br>Ahmedabad – 380 052, India<br>Phone: +91-79-2748 1415 / 5767

C. TORRENT RESEARCH CENTRE

**SIRT Expression analysis**	Dr Shailesh Deshpande<br>Cell and molecular Biology Lab,<br>Torrent Pharmaceuticals Limited<br>Torrent Research Centre<br>Dist Gandhinagar 382 428<br>Gujarat,India<br>Phone: +91-79-23969100 Ext. 715

1. TABLE OF CONTENTS

Investigator Signature Page 2
Sponsor Approval Signature Page 3
Sponsor Contact Details....4
Contact Details for Personnel at Study Site....6
Details of Study Facilities and Personnel 7
1. TABLE OF CONTENTS....9
2. SYNOPSIS....14
3. INTRODUCTION 19
3.1 Rationale for Current Study....25
4. OBJECTIVES 26
4.1 Primary Objective....26
4.2 Secondary Objectives 26
5. STUDY DESIGN....27
5.1 Overview 27
5.2 Rationale for Study Design, Control, Doses and Study Population 28
6. STUDY POPULATION AND Treatment....29
6.1 Number and Description of Subjects....29
6.2 Inclusion Criteria 29
6.3 Exclusion Criteria....30
6.4 Sample Size Calculation....31
6.5 Investigational product 31
6.6 Prior and Concomitant Medication....31
6.7 Restrictions on Subjects....32
6.7.1 Diet....32
6.7.2 Alcohol, caffeine, grapefruit....32
6.7.3 Exercise 32
7. INVESTIGATIONAL PRODUCT Administration 33
7.1 Description of investigational products....33
7.2 Summary of pharmaceutical, nonclinical and clinical information of TRC150094 33
7.2.1 Physical, Chemical & Pharmaceutical summary 33
7.2.2 Nonclinical Studies 33
7.2.3 Available Clinical Data....36
7.2.4 Summary of potential risks and benefits....39
7.2.5 Description and justification of route of administration and dosage39
7.3 Dose Administration....40
7.4 Supply, Identification and Storage 40
7.5 Treatment Compliance 41
7.6 Treatment of Overdose 42
7.7 Accountability 42

7.8 Dispensing of Investigational Product 42
8. METHODS 43
8.1 Study parameters/endpoints 43
8.1.1 Main study parameter/endpoint 43
8.1.2 Secondary study parameters/endpoints 43
8.2 Randomisation and treatment allocation 43
8.2.1 Preparation of randomisation code 43
8.2.2 Breaking the randomisation code 43
8.3 Blinding 44
8.4 Study Procedures 44
8.4.1 Hyperinsulinemic Euglycemic Clamp 44
8.4.2 **Quantification of Hepatic Fat.** 46
8.4.3 **Silent Information Regulator T (SIRT) expression study:** 47
8.4.4 **Measurement of Sagittal Abdominal diameter** 47
8.5 Study Visits 47
8.5.1 . Clinical laboratory tests 51
8.5.2 Vital signs 52
8.5.3 12-Lead ECGs 52
8.5.4 Physical examinations 52
8.5.5 Chest X ray 52
8.5.6 Laboratory investigations and bioanalysis of blood samples 52
8.5.7 Bioanalysis 54
8.5.8 Sample retention / destruction 54
8.6 Appropriateness of Measurements 54
8.7 Total Volume of Blood 54
8.8 Withdrawal Criteria 55
8.9 Withdrawal Procedures 55
8.10 Replacement of Dropout/Withdrawals 55
8.11 Follow up of subjects withdrawn from treatment 56
8.12 Study Termination 56
9. SAFETY Reporting 56
9.1 Safety and tolerability assessments and reporting 56
9.1.1 Safety Monitoring 56
9.1.2 Section 10 WMO event 56
9.1.3 Adverse events and serious adverse events 57
9.1.4 Reporting of adverse event 57
9.1.5 Procedures for reporting SAEs 58
9.1.6 Emergency procedures 59
9.1.7 Suspected unexpected serious adverse reactions (SUSAR) 59
9.1.8 Annual safety report 59
9.1.9 Follow-up of adverse events 60

10. DATA MANAGEMENT AND STATISTICAL ANALYSIS 61
10.1 Sample Size Calculation 61
10.2 Data Handling 61
10.3 Statistical Analysis 61
10.3.1 Descriptive statistics 61
10.3.2 Univariate analysis 61
10.4 Safety and Tolerability Analysis 62
11. ETHICAL AND REGULATORY CONSIDERATIONS 62
11.1 Regulation statement 62
11.2 Recruitment and consent 62
11.3 Benefits and risks assessment, group relatedness 63
11.4 Compensation for study related illness/injury 64
12. ADMINISTRATIVE ASPECTS AND PUBLICATION 64
12.1 Handling and storage of data and documents 64
12.2 Amendments 65
12.3 Annual progress report 66
12.4 End of study report 66
12.5 Public disclosure and publication policy 66
13. QUALITY CONTROL AND QUALITY ASSURANCE 67
14. REFERENCES 74

LIST OF TABLES

Table 1: Summary of Blood Volumes for Each Subject 54

LIST OF FIGURES

Figure 1: Pathologies originating form visceral adiposity and Insulin resistance 19
Figure 2: Concept of cardiometabolic risk 20

LIST OF APPENDICES

Appendix A: Clinical laboratory tests 67
Appendix B: Study visits 68
Appendix C: Flow chart of clamp procedure 71

LIST OF ABBREVIATIONS

AE	Adverse Event
ACE	Angiotensin Converting Enzyme
ALT	Alanine Transaminase
Apo B	Apolipoprotein B
aPTT	Activated Partial Thromboplastin Time
AST	Aspartate Transaminase
ASCVD	Atherosclerotic Cardiovascular Disease
AUC	Area Under the Curve
Od	Once daily
BMI	Body Mass Index
BUN	Blood Urea Nitrogen
C_{max}	Maximum observed plasma drug concentration
CVD	Cardiovascular Disease
CVS	Cardiovascular System
CHD	Coronary Heart Disease
CMR	Cardiometabolic Risk
CNS	Central Nervous System
CRF	Case Report Form
CRO	Clinical research organisation
CRU	Clinical Research Unit
CTA	Clinical Trial Application
CYPs	Cytochrome P
DIO	Diet Induced Obesity
ECG	Electrocardiogram
EP	Endogenous Production
eGFR	Estimated Glomerular Filtration Rate
FFA	Free Fatty Acid
fT3	Free T3
fT4	Free T4
GCP	Good Clinical Practice
GGT	Gamma Glutaryl Transferase
GI	GastroIntestinal
GMP	Good Manufacturing Practice
GST	Glutathione S-Transferase
HBsAg	Hepatitis B surface Antigen
HDL	High Density Lipoprotein
HED	Human Equivalent Dose
HIV	Human immunodeficiency virus
Hs CRP	Highly sensitive C-Reactive Protein
ICF	Informed consent form
ICH	International Conference on Harmonisation
IEC	Independent Ethics Committee
IL6	Interleukin 6
IMPD	Investigational Medicinal Product Dossier
IR	Insulin Resistance
IRB	Institutional Review Board
LC/MS/MS	Liquid Chromatography/Mass Spectrometry/Mass Spectrometry
LDL	Low Density Lipoprotein
Lp	Lipoprotein
LVP	Left Ventricular Pressure

MedDRA	Medical Dictionary for Regulatory Activities
MDRD	Modification of Diet in Renal Disease
MAD	Multiple Ascending Dose
MCP-1	Monocyte Chemotactic Protein 1
MI	Myocardial Infarction
IEC	Medical Research Ethics Committee
MRS	Magnetic Resonance Spectroscopy
RMR	Resting Metabolic Rate
MABEL	Minimal Anticipated Biological Effect Level
NOAEL	No-Observed-Adverse-Effect Level
NAFLD	Non-alcoholic Fatty Liver Disease
NEFA	Non-esterified Fatty Acid
Od	Once daily
PAI-1	Plasminogen Activator Inhibitor
PBMNC	Peripheral blood mono-nuclear cells
PCOD	Polycystic Ovary Disease
PCR	Polymerase Chain Reaction
PK	Pharmacokinetic
3-OHB	3-hydroxybutyrate
SCD-1	Stearoyl-CoA desaturase-1
PPAR- γ	Peroxisome Proliferator Activated Receptor- γ
QTc	Corrected QT
SAD	Single Ascending Dose
SAE	Serious Adverse Event
SIRT	Silent Information Regulator T
SUSAR	Suspected Unexpected Serious Adverse Reaction
T2	Diiodothyronine
T3	Triiodothyronine
T4	Thyroxine
TNF	Tumour Necrosis Factor
TR	Thyroid Receptor
TSH	Thyroid Stimulating Hormone
ULN	Upper Limit of Normal
VAT	Visceral Adipose Tissue
VLDL	Very Low Density Lipoprotein
WHO	World Health Organization
WMO	Wet Medisch-wetenschappelijk Onderzoek met Mensen (Medical Research Involving Human Subjects Act)

2. SYNOPSIS

**Sponsor:**<br>Torrent Pharmaceuticals Limited, Torrent Research Centre, Village Bhat, Dist. Gandhinagar, Gujarat, India.	
**Study number:**<br>CT/P015/CMR/2010/02_01	**Investigational medicinal product:**<br>TRC150094
**CTRI number:** REFCTRI-2010 002973	
**Title of study:**<br>A Phase 2A, Double-blind, Placebo-controlled, Randomized Study to Evaluate the Safety and Efficacy of TRC150094 in Increasing Insulin Sensitivity in Male patients with increased Cardiometabolic Risk.	
**Principal Investigators:**<br>1. Prof. E.S.G Stroes at Academic Medical Centre, Amsterdam, The Netherlands<br>2. Dr. Dharmesh Domadia at Veeda Clinical Research, Ahmedabad, India	
**Study centres:**<br>1. Academic Medical Centre, Amsterdam, The Netherlands<br>2. Veeda Clinical Research, Ahmedabad, India	
**Planned study period:**<br>6 months	**Clinical Phase:**<br>Phase 2A
**Objectives:**<br>Primary: To determine the safety and efficacy (in increasing insulin sensitivity) of TRC150094 once daily dosing for 4 weeks in male patients with increased cardiometabolic risk.<br><br>Secondary:<br>• To evaluate the effect of TRC150094 on hepatic fat and metabolic parameters.<br>• To evaluate the ethnic differences for effect of TRC150094 on Insulin sensitivity parameters	
**Study Design:**<br>This is a Phase 2A, two-centre, double-blind, randomized, placebo-controlled, multiple-dose, parallel study. Each subject will attend the study centre for 1 screening visit, 2 study visits (1 baseline and 1 end of treatment), 1 intermediate safety visit and 1 post-study follow-up visit (Total 5 visits). Total 40 subjects will take part in the study; 20 subjects will be enrolled in Ahmedabad, India and 20 will be enrolled in Amsterdam, Netherlands. Subjects will be randomized 1:1 for active treatment versus placebo. Dosing will take place for 28 days (Days 1–28).	
**Inclusion Criteria**<br>Subjects will be considered eligible for entry in the study if they meet all	

of the following criteria.

1. Adult male
2. Age range 30–65 years at screening
3. Caucasian or Indian ethnicity
4. Waist circumference ≥ 102 cm for Caucasians and ≥ 90 cm for Indians at screening.
5. Fasting Serum Insulin ≥ 10 mU/ml at screening
6. Blood Pressure ≥ 130/85 mmHg at screening (or patients taking medication for hypertension)
7. Stable weight during 3 months prior to the study (assessed through medical history of the patient)
8. Drug naive diabetic patients* or patients with impaired fasting glucose i.e > 100 mg/dl or 5.5 mmol/l and < 200 mg/dl or 11.0 mmol/l Diabetic patients who were taking metformin and have undergone washout for at least 4 weeks before Day 0 and are currently on life style modification as a treatment for diabetes will also be allowed in the study
9. Willingness to give written informed consent (prior to any study-related procedures being performed) and ability to adhere to the study restrictions and assessments schedule.

*** Diabetic patient is defined as a patient with a documented history of type II DM or a documented history of a fasting glucose > 200mg/dl or 11.0 mmol/l or 2x fasting glucose > 126 mg/dl or 6.9 mmol/l (2x =recorded twice).**

Exclusion Criteria

Subjects will not be considered eligible for entry in the study if they meet one or more of the following criteria.

1. Medical history, physical examination, vital signs, clinical laboratory tests, 12-lead ECG and Chest X ray (to exclude tuberculosis in India only) with any significant abnormalities, in the opinion of the investigator.
2. Subjects with any known somatic illness, including neoplasm, endocrine disorder such as cushing's disease, PCOD and uncontrolled hypothyroidism, neurologic disorder, active infection, or recent surgical procedure within 3 months of the study initiation.
3. Subject currently using medication, which can influence glucose or FFA metabolism such as fibrates, niacin, ACE inhibitors (subjects are not excluded in case of use of a stable dose for at least 6 weeks prior to baseline measurement, taking (ACE) inhibitors (ACE-I) or angiotensin-receptor blockers (ARBs)), PPAR agonists, omega 3 fatty acids.
4. eGFR < 60 mL/min/1.73m^2 at screening as evaluated by Modification of Diet in Renal Disease (MDRD) method.
5. History of angina, Myocardial Infarction (MI) or stroke since last 6

months.

6. Hypertension with SBP/DBP ≥160/100 mmHg at screening.
7. ALT or AST ≥ ULN*3 at screening
8. History or presence of malignancy.
9. History of recreational drug use within the last 30 days, or regular consumption of greater than 2 units of alcohol/day.
10. History of allergy to the test drug or any drug chemically similar to the drug under investigation.
11. Seropositive for Hepatitis B, Hepatitis C or HIV.
12. Subjects suffering from any psychiatric (acute or chronic) illness.
13. Intake of any medication except those permitted in this study (see Section 6.6).
14. Intake of any investigational drug in the period within 3 months prior to the first dose of study drug.
15. History of significant blood loss due to any reason, including blood donation, in the 12 weeks prior to the first dose of study drug; or the total blood loss in the last 3 months, including for this study, exceeds 450 mL.
16. History of any bleeding disorder.
17. Existence of any surgical or medical condition which, in the judgment of the principal investigator, might interfere with the absorption, distribution, metabolism or excretion of the study drug or might be likely to compromise the safety of the subject.
18. Inability to communicate or co-operate with the investigator because of language problems, poor mental development or impaired cerebral function.
19. Inability to comply with study requirements.
20. Positive drugs of abuse test (at screening) and alcohol breath test.
21. Heavy smokers (who are smoking >15 cigarettes or equivalent per day).

Test product, dose and mode of administration:

TRC150094 tablets, Tentative dose 50 mg (1 x 50 mg tablet)

Mode of administration: oral

Duration of treatment:

Once daily dosing on Days 1−28

Reference therapy, dose and mode of administration:

Placebo tablets for oral administration (identical taste, appearance to TRC150094 tablets)

Criteria for evaluation:

Insulin Sensitivity:

- Rate of Glucose Disposal
- Suppression of Endogenous Glucose Production

- Suppression of rate of lipolysis

Early efficacy markers that will be explored include:

- Hepatic fat
- Lipid parameters
- Metabolic markers

Methodology

On screening, patients will visit the trial unit after an overnight fast. After informed consent is obtained, subjects will be screened for eligibility to participate in the study. Screening will include a careful assessment of the inclusion/exclusion criteria and collection of blood and urine for screening tests. Subjects will give a full medical history, and undergo a full physical examination, including height, body weight, waist circumference and vital sign measurements. A 12-lead electrocardiogram (ECG) tracing will be obtained to screen for underlying cardiac disease. All subjects will receive life style instructions (normal diet, ≤ 2 alcohol consumptions a day, restriction on caffeine and grapefruit intake, no strenuous or unaccustomed exercise). If a patient is on oral metformin treatment, treatment will be discontinued until the end of the study. Subjects who meet all entry criteria will be eligible for randomization on Day 0.

On Day 0 patients will visit the trial unit after an overnight fast. The patients may be asked to present in CRU on Day -1 if required (for baseline investigations). At Day 0 (and/or Day -1), the following assessments will be performed: Physical examination, vital signs, height and body weight, waist circumference, clinical laboratory tests, urine collection (urinalysis), 12-lead ECG, AEs and change in concomitant medications will be reported, hyperinsulinemic euglycemic clamp (the clamp procedure is described in Appendix C) will be performed. Liver fat will be measured by 1H MRS. All patients will get life style instructions.

On day14±1, the patients will visit the trial unit after an overnight fast. The following assessments will be performed on day 14±1: vital signs, height and body weight, waist circumference, safety clinical laboratory tests, urine collection (urinalysis), 12-lead ECG, AEs and change in concomitant medications will be reported, all patients will get life style instructions.

On Day 28+2, patients will visit the trial unit after an overnight fast. At Day 28, the following assessments will be performed: physical examination, vital signs, height and body weight, waist circumference, clinical laboratory tests, urine collection (urinalysis), 12-lead ECG, AEs and change in concomitant medications will be reported, hyperinsulinemic euglycemic clamp (the clamp procedure is described in Appendix C) will be performed, liver fat will be measured by 1H MRS, discontinuation of study drug, all patients will get life style instructions.

At Day 35-38, patients will visit the trial unit after an overnight fast and the following assessments will be performed: physical examination, vital signs, height and body weight, waist circumference, safety clinical laboratory tests, urine collection (urinalysis), 12-lead ECG, AEs and change in concomitant medications will be reported, restart of oral metformin treatment (if applicable).

For subjects who are withdrawn, the above assessments should be performed within 8-10 days after the last drug administration.

Benefit and risk

This study does not have specific advantages for the study subjects. The results of this study may help researchers learn whether TRC150094 may be beneficial for the treatment of male subjects with increased cardiometabolic risk associated with non-traditional risk factors. [6,6-2H2] glucose is glucose labeled with a stable isotope of hydrogen, which behaves as the natural substrate and has no side effects. [1,1,2,3,3-2H2] labelled glycerol is glycerol labeled with 5 stable isotope of hydrogen, which behaves as the natural substrate and has no side effects. Actrapid is fast acting insulin, a hormone that could induce hypoglycemia. However, it is not in the scope of this protocol to allow hypoglycemia to occur, since plasma glucose concentration will be fixed at 5 mmol/l by a variable infusion of glucose 20% guided by frequent bedside glucose measurements. An overview of the blood samples and blood volumes taken during the study is provided in Table 1. The total blood volume to be withdrawn from any individual subject will be 391.2 ml for subjects at Veeda Clinical Research, India and 361.2 ml at AMC, Netherlands. This amount is not considered to be of negative influence to the subject's health. A possible side effect of clamping, which is very rare and mostly preventable, could be hypoglycaemia. MR- spectroscopy of the liver will be made which takes about 30 minutes. This spectroscopy is not considered to be potentially harmful to subjects.

3. INTRODUCTION

Background

A plethora of studies have shown that visceral adiposity and insulin resistance are the key underlying factors associated with clustering atherogenic abnormalities which include a typical atherogenic dyslipidemic state (high triglyceride and apolipoprotein B concentrations, an increased proportion of small dense LDL particles and a reduced concentration of HDL-cholesterol with HDL particles also being smaller in size), a prothrombotic profile, and a state of inflammation. Furthermore, visceral adiposity and insulin resistance could also contribute to an elevated blood pressure and to dysglycemia[1] eventually leading to Atherogenic Cardiovascular Disease[2,3,4] and Type 2 diabetes[5,6] Fig 1 describes the pathologies originating from Visceral adiposity and Insulin Resistance.

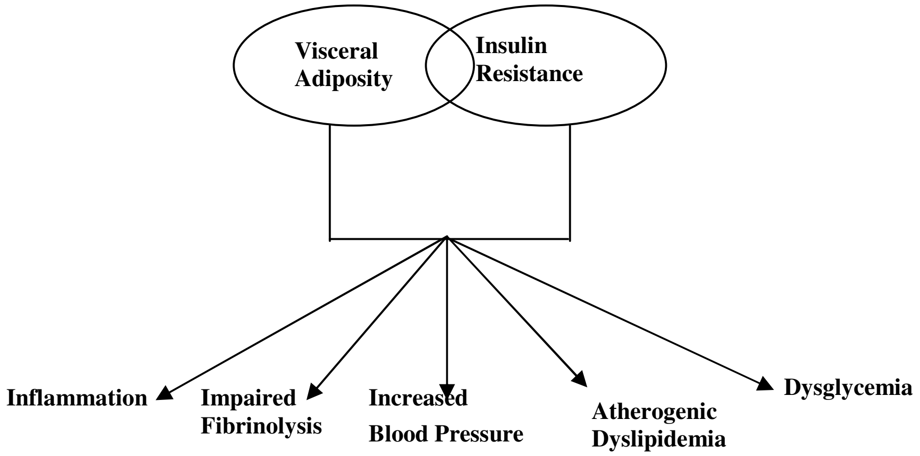

Fig 1: Pathologies originating from Visceral Adiposity and Insulin Resistance

The incidence of visceral adiposity and the related comorbidities are increasing worldwide, leading to increased burden of cardiovascular risk in general population. Traditional risk factors as defined by Framingham Heart Study (age, race, gender, smoking, hypertension, High Cholesterol and family history) do not completely account for the cardiovascular risk in majority of population. Non-traditional risk factors (also known as Non-framingham risk factors) such as obesity, insulin resistance, dyslipidemia, dysglycemia and Hypertension are equally important culprit for increase in CV risk. Hence Cardiometabolic risk which is the overall risk of

cardiovascular disease (CVD) and diabetes resulting from the presence of the non-traditional risk factors and also of traditional risk has been described as a plausible target for treatment[1]. Figure 2 elucidates the concept of Cardiometabolic risk.

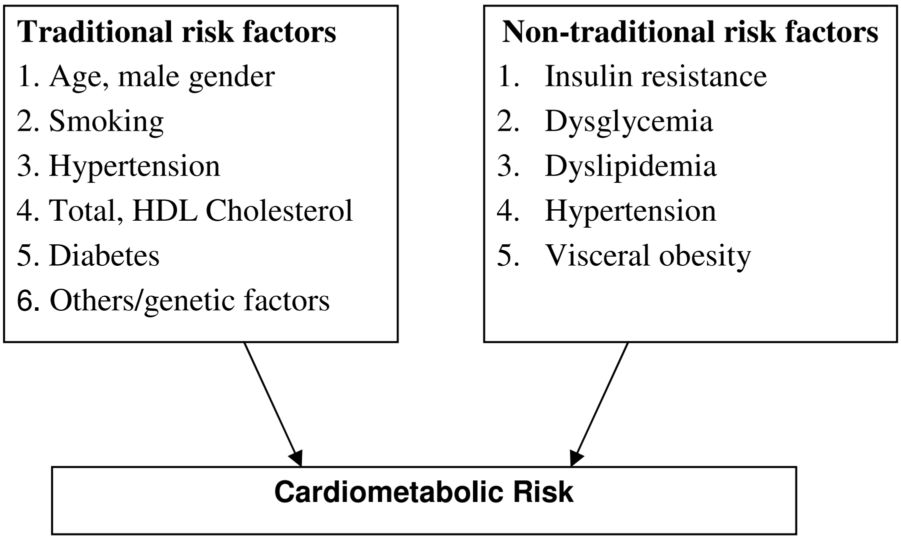

Fig 2: Concept of Cardiometabolic Risk

The pathophysiology underlying Cardiometabolic risk is complex and has been only partially elucidated.

There is considerable evidence supporting the notion that excess abdominal fat is predictive of insulin resistance and the presence of related metabolic abnormalities. Studies of the measurement of abdominal adiposity (magnetic resonance imaging and computed tomography) have commonly reached the conclusion that the amount of visceral adipose fat not that of subcutaneous abdominal fat, is a quite dominant correlate of metabolic abnormalities observed in overweight/obese patients

Visceral Adipose tissue (VAT) is not only an energy storage tissue, but also a metabolically active organ secreting hormones, cytokines and growth factors, collectively called as adipocytokines. It is believed that anti-atherosclerotic adipocytokines, such as leptin and adiponectin, and proatherosclerotic cytokines, such as interleukin (IL)-6 and tumor necrosis factor (TNF)-α, cooperatively regulate metabolic and cardiovascular homeostasis at local and remote sites. Visceral adiposity perturbs this homeostasis thus resulting in pro-atherogenic state.[7]

Visceral adiposity is also associated with a pro-inflammatory state which is also an etiological factor for the atherosclerotic process. Overall, these pathophysiological

processes culminate into increased risk of ASCVD (Atherosclerotic Cardiovascular Disease).[2,3,4].

Visceral adiposity is an important causative factor for whole body Insulin resistance. Visceral adiposity leads to increased fatty acid load to liver and muscle forming an Insulin resistant milieu in liver and muscle tissues.[1]

Visceral adiposity and associated insulin resistance contributes to exposure of high concentrations of free fatty acids (due to increased lipolysis) to the liver (through the portal circulation), impairing several hepatic metabolic processes leading to hyperinsulinemia (decreased insulin clearance), glucose intolerance (increased hepatic glucose production), and hypertriglyceridemia (increased VLDL-apolipoprotein B secretion).[1]

Intra-hepatic fat thus represents an important contributor to the pathogenesis of Cardiometabolic risk.

In fact various global studies (eg The Framingham Heart Study) have shown that adiposity represented by increased intrahepatic fat (IHF), compared with general adiposity (represented by BMI), would explain more of the variance in cardiovascular disease (CVD) risk factors.[8]

Insulin resistance (IR) is another important component of cardiometabolic risk and consists of two important features; peripheral and hepatic insulin sensitivity. Peripheral insulin resistance leads to decreased insulin stimulated glucose uptake in skeletal muscle and adipocytes. Hepatic IR increases plasma glucose levels by decreasing insulin mediated suppression of glucose production. The physiological mechanisms underlying insulin resistance are complex and not fully elucidated. Amongst others, an impairment of the skeletal muscle metabolic energy transduction pathways (mitochondrial dysfunction) has been suggested to be casually related to insulin resistance. Mitochondrial fatty acid overload and incomplete fatty acid oxidation have been suggested to result in increased availability of fatty acids for lipid accumulation. Fatty acid metabolites are thought to induce insulin resistance in both liver and muscle by impairing the insulin-signaling pathway.[9]

Evidences suggest that Visceral adiposity, one of the most important cardiometabolic risk factors originates from a chronic imbalance between energy intake and expenditure.[10]

At the cellular level, various pathophysiological events responsible for metabolic abnormalities have been hypothesized. Among these, impaired cellular metabolism has been suggested as a key pathophysiological process. In particular, defective oxidative metabolism suggesting impairment in mitochondrial function. seems to be involved in visceral fat gain and in the development of insulin resistance[11], Evidences are emerging to support the concept that alterations in mitochondrial biogenesis and energetics are linked to the development and progression of metabolic disorders and CVD in obesity.[12, 13]

Both obesity and common forms of type-2 diabetes have been found to be associated with reduction in mitochondrial size and number, which in turn results into reduced activity of tricarboxylic acid cycle, E- oxidation, electron transport enzymes and glucose oxidation. In diabetes mellitus, a potential role of deregulation of expression of genes involved in oxidative metabolism has also been suggested by studies in animals and human.[14-17].

It has also been hypothesized that the abnormally high availability of fat as a substrate for energy leads to reduction in oxidative capacity of mitochondria and hyperinsulinemia.[18]

This vicious cycle of lower energy expenditure leading to accumulation of fat and further reduction of oxidative capacity of mitochondria eventually culminates into visceral obesity, insulin resistance, hypertension, impaired lipid profile, thus overall increased cardiometabolic risk.

There is clearly a significant unmet medical need for safe and effective weight-reducing therapies to prevent the debilitating metabolic diseases and mortality that are associated with increasing central adiposity.

To date, pharmacological agents for the management of Cardiometabolic risk have been limited and unsatisfactory. Most have attempted to address weight reduction by targeting appetite at the central nervous system.[19] So far most of these with central effect have been plagued by a higher incidence of depressive symptoms or have resulted in higher incidence of cardiovascular side-effects.

Thyroid hormones (THs) play an important role in several physiological processes including growth and development in early life as well as metabolic control later in life. The mechanism underlying the calorigenic effects of THs remains to be elucidated. Among the widespread actions of thyroid hormones (THs), increasing

metabolic rate[20] and lowering atherogenic serum lipoproteins are clinically interesting metabolic responses since these could hypothetically be of use to treat obesity and its related co-morbidities[21, 22]. However previous attempts to mimic the effects of THs using thyroid hormone metabolites and analogues have been complicated by the induction of thyrotoxic adverse effects in other organ systems such as the heart and skeleton[23, 24]. Consequently, the development of a TH agonist or analogue that retains anti obesity efficacy while being devoid of thyrotoxic effects, would represent a potentially valuable therapeutic approach for obesity related co-morbidity. T2 (3.5-diiodothyronine) is a metabolically active iodothyronine with the capacity to stimulate oxidation of fatty acids in metabolically active tissues (heart, skeletal muscle, liver and Brown adipose tissue) at relatively low concentrations. T2 has lesser affinity towards the thyroid receptor compared to T3 and its metabolic effects are rapid [25].

TRC150094 is a novel thyromimetic analogous to 3.5-diiodothyronine (T2) being developed by Torrent for the treatment of Cardiometabolic risk associated with non-traditional risk factors.

TRC150094 is a thyromimetic analogous to T2 that aims at increasing the energy expenditure and thus regaining the balance.

In various preclinical studies conducted in multiple animal models of visceral adiposity, insulin resistance and metabolic syndrome, it was found that TRC150094 increases energy expenditure through increase in mitochondrial metabolic activity and attenuates visceral adiposity, atherogenic dyslipidemia, blood pressure and improved insulin sensitivity.

If these effects are replicated in clinical setting, this profile will prove invaluable in the treatment of Cardiometabolic risk associated with visceral adiposity.

Clinically, the continuous administration of TRC150094 administration leading to sustained increase in Energy Expenditure is expected to result in:

- Decrease in adiposity leading to a more desirable body mass composition,
- Decrease in hepatic fat thus improvement in NAFLD,

- Improvement in insulin sensitivity thus improvement in glucose homeostasis,

- Improvement in insulin sensitivity, dependent renal-hormonal axis (RAAS and SNS), endothelial function and thus, improvement in Blood Pressure profile,

- Improvement in lipid profile.

Phase 1 Single Dose studies have been completed that included Single Ascending dose study in overweight/obese subjects, food effect study and elderly study. During these studies, TRC150094 has been found to be well tolerated; with safety established up to 400 mg single dose in overweight/ obese subjects of age group 18-65 years. Multiple Ascending Dose study is ongoing. There were two sequential dose levels (50 mg OD and 150 mg OD for 28 days) planned for this study. Of these, the first cohort (50 mg OD for 28 days) has been completed and the safety has been established on this dose.

3.1 Rationale for Current Study

Insulin resistance (IR) is a major factor responsible for Cardiometabolic risk and consists of two important features: peripheral and hepatic insulin resistance. Peripheral insulin resistance leads to decreased insulin stimulated glucose uptake in skeletal muscle and adipocytes as well as decreased insulin-mediated suppression of free fatty acids (FFA)-release. Hepatic insulin resistance increases plasma glucose levels by decreasing insulin mediated suppression of glucose production. The physiological mechanisms underlying insulin resistance are complex and not fully elucidated. Amongst others, an impairment of the skeletal muscle metabolic energy transduction pathways (mitochondrial dysfunction) has been suggested to be causally related to insulin resistance[9] Mitochondrial and cytosolic fatty acid overload and incomplete fatty acid oxidation have been suggested to result in increased availability of fatty acids for lipid accumulation. Fatty acid metabolites are thought to induce insulin resistance in both liver and muscle by impairing the insulin-signaling pathway. Since T2 has been shown to activate processes enhancing fatty acid oxidation and thermogenesis, T2 could play a role in decreasing fat accumulation in muscle and liver and improve insulin sensitivity. Indeed TRC150094 has been shown to improve insulin sensitivity in preclinical studies. Whether TRC150094 will also improve insulin resistance in clinical studies remains to be elucidated. We therefore propose to investigate the safety and efficacy of treatment with TRC 150094 on increasing peripheral and hepatic insulin sensitivity as well as lowering muscle and liver triglyceride concentration in patients with the metabolic syndrome. The current Phase 2A study is designed to investigate safety and efficacy of TRC150094 for increasing insulin sensitivity in patients with increased cardiometabolic risk. This study will be a double-blind, randomized, placebo-controlled, multiple-dose, parallel study to assess effect on insulin sensitivity and to explore early efficacy markers (hepatic fat, lipid parameters and metabolic markers) after oral administration of TRC150094 tablet for 28 days. The tentative dose will be 50mg OD. This dose level has been calculated based on the assessment of safety and PK data obtained from SAD study. The exact dose level will be confirmed on the basis of interim safety and PK data obtained from MAD study. Full discussion of the study design is presented in Section 5.

4. OBJECTIVES

4.1 Primary Objective

To determine the safety and efficacy (in increasing insulin sensitivity) of TRC150094 once daily dosing for 4 weeks in male patients with increased cardiometabolic risk.

4.2 Secondary Objectives

- To evaluate the effect of TRC150094 on hepatic fat and metabolic parameters.
- To evaluate the ethnic differences for effect of TRC 150094 on Insulin sensitivity parameters

5. STUDY DESIGN

5.1 Overview

This will be a Phase 2A, two-centre, double-blind, placebo-controlled, multiple-dose study to assess the effect of multiple oral doses of TRC150094 on insulin sensitivity in 40 overweight/obese male subjects. 20 Subjects will be enrolled in India and another 20 subjects at Amsterdam, the Netherlands. The maximum duration of participation in the study for each subject will be 17.5 weeks including a ≤12 weeks screening period, 4 weeks of treatment and a 10 days post treatment follow-up evaluation period (see Appendix B for Study Assessments).

At each study site 20 subjects will be enrolled. Each subject will attend the study centre in a fasting state, for a screening visit, 2 study visits (one baseline and one end of treatment), 1 intermediate safety visit and 1 post-study follow-up visit (Total 5 visits). The subjects at each site will be randomized to receive TRC150094 or placebo in a ratio of 1:1. The tentative dose level is 50 mg to be administered OD (morning) under fasting conditions. Dosing will take place daily on Days 1−28. Subjects will arrive at the study centre for screening visit. Physical examination, vital signs, safety biochemistry and laboratory investigations for verification of inclusion/ exclusion criteria will be performed during screening visit. Subjects meeting all the inclusion criteria and none of the exclusion criteria and who have given their informed consent for the study will be asked to come for the study on Day 0 (or day -1 if required). Baseline investigations (including baseline clamp procedure and hepatic MRS) will be done on Day 0 (or day -1). Subjects will receive properly labelled bottle containing either Active treatment or Placebo as per the randomization number of the subject. Subjects will be asked to take the dose (tentatively 50mg) OD in the morning on Days 1-28 inclusive, in fasting conditions, with a glass of water. Drug compliance of at least 90% will be ensured. Hence the allowable limit of missing the dose should not be more than 3 days in total. Discontinuation should not be of more than 2 consecutive days at any point of time. Uniformity in timing of intake of medication will be advised which should be preferably within ± 1 hr of the time of intake of study medication on Day 1. Each subject will attend the study centre on Day 14 for safety investigations. A deviation of ± 1 day will be allowed for these visits. Subjects will attend the study centre again on Day 28 for clamp procedure and hepatic MRS. A

deviation up to +2 days is acceptable only in those cases that missed doses within the limits given above and has continued planned medication up to day of post-dose investigations. For further details, see Section 8.4 and Appendix A. It is planned that the study will take place over 6 months (including screening and follow-up periods).

5.2 Rationale for Study Design, Control, Doses and Study Population

The tentative dose (50 mg) has been selected based on estimates of safety from pre-clinical studies, and safety and tolerability data obtained from SAD study. The study will begin subject to the availability of safety data of relevant dose of MAD study (currently ongoing). A placebo control has been included in the study design to allow for an unbiased assessment of insulin sensitivity. The study will be double-blind and randomized to ensure unbiased data.

6. STUDY POPULATION AND TREATMENT

6.1 Number and Description of Subjects

It is intended that 40 subjects complete the study: 20 subjects in India and 20 subjects in Amsterdam, the Netherlands. An attempt will be made to dose 20 subjects at each study site (10 for Active treatment; 10 for Placebo treatment). Subjects will be identified by their initials. In addition, subjects will be given a screening number at screening and a subject (randomisation) number at the time of randomisation. A list identifying the subjects by initials, screening number, subject number and status (completed study/withdrawn) will be kept in the trial master file.

6.2 Inclusion Criteria

Subjects will be considered eligible for entry in the study if they meet all of the following criteria.

1. Adult male
2. Age range 30–65 years at screening
3. Caucasian or Indian Ethnicity
4. Waist circumference ≥ 102 cm for Caucasians and ≥ 90 cm for Indians at screening.[27]
5. Fasting Serum Insulin ≥ 10 mU/ml at screening
6. Blood Pressure ≥ 130/85 mmHg at screening(or patients taking medication for hypertension)
7. Stable weight during 3 months prior to the study (assessed through medical history of the patient)
8. Drug naïve diabetic patients* or patients with impaired fasting glucose i.e > 100 mg/dl or 5.5 mmol/l and < 200 mg/dl or 11.0 mmol/l. Diabetic patients who were taking metformin and have undergone washout for at least 4 weeks before Day 0 and are currently on life style modification as a treatment for diabetes will also be allowed in the study
9. Willingness to give written informed consent (prior to any study-related procedures being performed) and ability to adhere to the study restrictions and assessments schedule.

*** Diabetic patient is defined as a patient with a documented history of type II DM or a documented history of a fasting glucose > 200 mg/dl or 11.0 mmol/l or 2x fasting glucose > 126 mg/dl or 6.9 mmol/l (2x = recorded twice)**

6.3 Exclusion Criteria

Subjects will not be considered eligible for entry in the study if they meet one or more of the following criteria.

1. Medical history, physical examination, vital signs, clinical laboratory tests, 12-lead ECG and Chest X ray (in India only) with any significant abnormalities, in the opinion of the investigator.
2. Subjects with any known somatic illness, including neoplasm, endocrine disorder such as cushing's disease, PCOD and uncontrolled hypothyroidism, neurologic disorder, active infection, or recent surgical procedure within 3 months of the study initiation.
3. Subject currently using medication, which can influence glucose or FFA metabolism such as fibrates, niacin, ACE inhibitors (subjects are not excluded in case of use of a stable dose for at least 6 weeks prior to baseline measurement, taking (ACE) inhibitors (ACE-I) or angiotensin-receptor blockers (ARBs)), PPAR agonists, omega 3 fatty acids.
4. eGFR < 60 mL/min/1.73m2 at screening as evaluated by Modification of Diet in Renal Disease (MDRD) method.
5. History of angina, Myocardial Infarction (MI) or stroke since last 6 months.
6. Hypertension with SBP/DBP ≥160/100 mm Hg at screening.
7. ALT or AST ≥ ULN*3 at screening
8. History or presence of malignancy.
9. History of recreational drug use within the last 30 days, or regular consumption of greater than 2 units of alcohol/day
10. History of allergy to the test drug or any drug chemically similar to the drug under investigation.
11. Seropositive for Hepatitis B, Hepatitis C or HIV.
12. Subjects suffering from any psychiatric (acute or chronic) illness.
13. Intake of any medication except those permitted in this study (see Section 6.6).
14. Intake of any investigational drug in the period within 3 months prior to the first dose of study drug.

15. History of significant blood loss due to any reason, including blood donation, in the 12 weeks prior to the first dose of study drug; or the total blood loss in the last 3 months, including for this study, exceeds 450 mL.
16. History of any bleeding disorder.
17. Existence of any surgical or medical condition which, in the judgment of the principal investigator, might interfere with the absorption, distribution, metabolism or excretion of the study drug or might be likely to compromise the safety of the subject.
18. Inability to communicate or co-operate with the investigator because of language problems, poor mental development or impaired cerebral function.
19. Inability to comply with study requirements.
20. Positive drugs of abuse test (at screening) and alcohol breath test.
21. Heavy smokers (who are smoking >15 cigarettes or equivalent per day).

6.4 Sample Size Calculation

It is intended that 40 subjects (20 subjects in India and other 20 in Amsterdam, the Netherlands) will complete the study. No formal sample size calculation has been performed. We calculated that a sample size of 20 in each group will have 80% power to detect an absolute difference in Rd of at least 15 μmol/kg·min (measure for peripheral insulin sensitivity), before and after treatment, using a Wilcoxon (Mann-Whitney) rank-sum test with a 0.05 two-sided significance level and assuming that the common standard deviation is 15.

6.5 Investigational product

Subjects will be treated with either TRC150094, or a placebo for 28 days. TRC150094 is a novel synthetic compound, chemically, 3-[4-(7-Hydroxy-6-methyl-indan-4-ylmethyl)-3,5-dimethyl-pyrazol-1-yl]-propionic acid, which is currently in clinical development for the treatment of Increased Cardiometabolic risk associated with non-traditional risk factors.

6.6 Prior and Concomitant Medication

Subjects are not permitted to enter the study if they have taken any investigational drug in the 3 months prior to study drug administration. Subjects are not permitted to enter the study if they have taken insulin or any oral anti-diabetic (except metformin)

in past. Those subjects who are taking metformin may be included in the study after a washout of at least 4 weeks. During these 4 weeks, these subjects should be on lifestyle modification as a treatment for diabetes. Other concomitant medications may be allowed after agreement between principal investigator and sponsor. Prior medications (up to 4 weeks prior to the dosing occasion) and all concomitant medications (up to the post-study follow-up visit) will be recorded in the subject's case report form (CRF).

6.7 Restrictions on Subjects

6.7.1 Diet

All subjects will fast for at least 10 hrs before Screening. Subjects will fast for approximately 13 hrs before Day 0 and Day 28 investigations. During the dosing period, the subjects will be instructed to fast for at least 1 hr prior to dosing (morning) and for 1 hr post-dose. Subjects are required to present to the research centre after an overnight fast of at least 10 hrs for Day 14±1 and follow-up visits.

6.7.2 Alcohol, caffeine, grapefruit

Subjects are not permitted any alcohol or caffeine-containing food or drinks from 48 hrs prior to study investigation (Day 0) until discharge before dosing period and similarly, from 48 hrs prior to post dose investigation (Day 28) until discharge. In addition, no alcohol is permitted for 48 hrs before the screening, Day 14±1 and follow-up visits. While resident in the CRU, all drinks and food will be decaffeinated. Subjects are permitted up to 6 caffeinated drinks per day in the period from screening to 48 hrs prior to admission for baseline investigation, during Day 1 to Day 26 and from discharge to the follow-up visit. Subjects are not permitted any grapefruit or grapefruit-containing food or drinks from 1 week prior to dosing (first study drug administration) until the post-study follow-up visit.

6.7.3 Exercise

Subjects must refrain from strenuous exercise for 48 hrs prior to the screening visit. Subjects must not undertake strenuous and/or unaccustomed exercise from 1 week prior to study investigation (Day 0). They are however allowed to undertake mild and accustomed exercise during their period of dosing (Day 1 to Day 28). Subjects must also refrain from strenuous and/or unaccustomed exercise during the period between discharge and follow-up visit.

7. INVESTIGATIONAL PRODUCT ADMINISTRATION

7.1 Description of investigational products

Active study drug:

Laboratory Code: TRC150094.

Excipients: Cellulose Microcrystalline, Hypromellose, Magnesium stearate

Formulation: Tablet

Strengths: 2.5 mg, 12.5 mg, 50 mg

Route of administration: Oral

Placebo tablets:

Contents: Cellulose Microcrystalline, Magnesium stearate

Formulation: Tablet

Route of administration: Oral

Placebo tablet will be administered orally (tablet will have an identical appearance and volume to TRC150094 tablet).

7.2 Summary of pharmaceutical, nonclinical and clinical information of TRC150094

7.2.1 Physical, Chemical & Pharmaceutical summary

TRC150094 is white to off-white powder, soluble in DMSO and practically insoluble in water. TRC150094 Tablets are white to off-white, round, uncoated tablets. TRC150094 tablets (all strengths) should be stored at room temperature not exceeding 25°C and protected from direct sunlight.

7.2.2 Nonclinical Studies

Pharmacology

To assess the potential of TRC150094 for the treatment of adiposity associated metabolic disorder, it was administered to diet induced obese C57BL6 mice (DIO mice), which simulate the pathophysiological features of human metabolic disorder.

The study revealed dose dependent increase in oxygen consumption and significant attenuation of body weight gain in TRC150094 treated group. Mice treated with TRC150094 had significantly ($p<0.05$) less abdominal, epididymal, perirenal, scapular, lumbar and total fat pad in comparison to control. TRC150094 treated group also showed significantly reduced LDL-C as compared to control. Fasting plasma insulin and glucose levels were estimated and HOMA-IR index was derived. HOMA-IR index was found to be lower in TRC150094 treated group as compared to control, thus indicating improved insulin sensitivity. TRC150094 has also shown to significantly reduce hepatic fat content as compared to control. Thus TRC150094 has shown beneficial effect on the individual components of metabolic syndrome that is adiposity, insulin sensitivity, lipid profile and hepatic fat in DIO mice. In another 4 week TRC150094 study on wistar rats fed on High fat diet, it was found that the treatment group had significantly less visceral adipose tissue than control. In liver, mitochondrial fatty acid import and oxidation were increased as compared to control, and consequently the hepatic triglyceride content was lower. These effects were independent of the AMP-activated protein kinase-acetyl CoA-carboxylase-malonyl CoA pathway but involved sirtuin-1 activation. In skeletal muscle, TRC induced a fiber shift toward the oxidative type in tibialis anterior muscle, increasing its capacity to oxidize fatty acids. HFD-TRC rats had lower (vs. HFD rats) plasma cholesterol and triglyceride concentrations.[26] Long term in vivo efficacy studies of TRC150094 were conducted using different animal models such as DIO mice, hamster and Ob ZSF1 rat. In the long term treatment studies, TRC150094 not only attenuated the visceral fat accumulation but also attenuated various cardiovascular risk components associated with visceral adiposity and type 2 diabetes. For instance, long term treatment of obese ZSF1 rat with TRC150094 improved glucose tolerance, glycemic and lipid profile, attenuated rise in blood pressure and improved functional capacity of skeletal muscle without influencing appetite, thyroid hormone, cardiac function and skeleton. Selectivity and safety pharmacology studies suggest that TRC150094 does not have significant enzyme inhibition or ligand binding activity towards a variety of enzymes and receptors. TRC150094 demonstrated no significant safety concerns in nonclinical cardiovascular, respiratory and CNS safety pharmacology studies and demonstrated a wide safety margin.

Pharmacokinetics and Metabolism

The nonclinical pharmacokinetic and metabolic profile of TRC150094 has been characterized. When administered orally, TRC150094 is rapidly absorbed in male Wistar rat with an absolute bioavailability of 92%. In toxicokinetic studies the Cmax achieved at 1-4 hrs in beagle dogs. TRC150094 has shown dose related increase in exposure at several fold higher pharmacological doses across the species and genders. The parent compound showed rapid and wide distribution. TRC150094 is metabolically stable in microsomes and hepatocytes of various species tested. There is no inhibition and induction of the major CYP450 enzymes at pharmacologically relevant concentrations. In vivo studies in rat revealed three Phase 2 metabolites in the form of hydroxylate, sulphate and glucuronate conjugates of the drug. TRC150094 has shown dose proportionality up to several folds of pharmacological doses across the species and genders, without any accumulation potential. The terminal half life of TRC150094 is 4-8 hrs in rats and 7-10 hrs in dogs. About 98% of the compound is excreted in 48 hrs through urine and faeces in rats.

Toxicology

TRC150094 was found to be non-mutagenic in AMES test, Mouse lymphoma assay and Micronucleus test. TRC150094 was found to be well tolerated in acute studies in rats and mice by clinical and parenteral route. Chronic repeat dose toxicity studies were conducted in Wistar rats (26 week) and Beagle dogs (39 week). There were no test item related clinical signs observed in rat and dog treated chronically. However, Beagle dogs treated in 4 week study showed emesis at 2-4 hr post dosing at high dose of 450 mg/kg/day. Transient reduction in thyroid hormones (total and free T4) was observed in rat studies at top doses of 180 and 360 mg/kg/day, while in dog it was seen in 4 week study at top dose of 450 mg/kg/day. Thyroid hormone profile remained unaltered in 39 week dog toxicity study up to a high dose of 75 mg/kg/day. The change in thyroid hormone profile is considered as test item related pharmacological effect. Repeated exposure of TRC150094 revealed kidney as target organ for toxicity in both rat and dog species. Increased kidney weight was evident at highest doses up to the chronic duration in rats. Microscopically, dilated renal tubules and nephropathy was seen at highest doses of 180 and 360 mg/kg/day in rats. Beagle dogs treated in 4 week study at 450 mg/kg/day revealed dilated renal tubules, lymphocytic infiltration and nephropathy in kidney and degeneration of seminiferous

tubules of testes. There were no such alterations observed in dog treated at high dose of 75 mg/kg/day in 13 and 39 week studies. No observed adverse effect level (NOAEL) doses in Wistar rat and Beagle dog is 60 and 75 mg/kg/day, respectively in chronic toxicity studies which demonstrated very high safety margins. TRC150094 did not have any adverse impact on male and female fertility indices studied in rats and found to be non-teratogenic in rat and rabbits.

7.2.3 Available Clinical Data

The clinical evaluation of TRC150094 to date consists of Single Dose studies and Cohort 1 of Multiple Ascending Dose Study.

Single Dose Studies:

The Single Dose Studies included:

PART A: Single ascending dose in non-elderly and elderly overweight/obese male and female subjects to evaluate safety, tolerability and pharmacokinetics of TRC150094.

PART B: To evaluate effect of food on pharmacokinetics of TRC150094 in young healthy male and female subjects and to assess the safety and tolerability of single oral doses of TRC150094 administered in fasted and fed states.

A total of 40 subjects were randomized in Part A. Out of 40 subjects, 24 were non-elderly overweight/obese subjects aged 18-65 years and 16 were elderly aged above 65 years. 24 subjects were divided in 3 cohorts (Cohort 1: 5 and 100mg; Cohort 2: 25 and 200 mg; Cohort 3: 50 and 400 mg) each having 8 subjects and 16 elderly subjects were assigned to fourth cohort (50 and 150 mg). Out of 24 non-elderly subjects, 18 were administered TRC150094 and 6 were administered placebo. Among elderly subjects, 12 were administered TRC150094 and 4 were administered placebo. Each subject received two dose levels with a washout period of 7 days in between the two study periods. In Part B of the study 6 healthy subjects aged 18-65 years were randomized to receive the active dose of 100 mg in fasting and fed conditions, with a washout period of atleast 7 days between the two study periods.

The main findings of these studies are as follows:

Safety and tolerability- The ascending doses of TRC150094 ranging from 5 mg to 400 mg, evaluated in the study were found to be safe and well tolerated in both elderly, as

well as non-elderly overweight/obese subjects. There were no SAEs or withdrawals due to AE in the study. All the adverse events were mild to moderate in nature. In non-elderly cohort, headache was the most common AE experienced by 3 (8.3%) subjects followed by decreased appetite, pain in extremity and dizziness postural, each experienced by 2 (5.6%) subjects in the TRC150094 group. Headache was the most common AE experienced by 2 (8.3%) subjects in the elderly cohort, also, followed by back pain experienced by 1 (4.2%) subject receiving TRC150094. No AE was reported in the placebo group in elderly cohort.

Pharmacokinetics- Absorption of TRC150094 was rapid (Tmax reached within 3 hrs) and elimination half life ranged from 15-18 hrs. AUC0-∞ was found to be dose proportional and Cmax seemed greater than dose proportional across the dose range. More than 80% of total excreted amount (during 48 hrs) in urine was recovered by 24 hrs in non-elderly and elderly subjects. Total exposure was found to be higher in elderly subjects compared to non-elderly subjects. Renal clearance has been observed to be decreased in elderly subjects as compared to non-elderly subjects. Gender related differences with Cmax and $AUC_{0-\infty}$ were not found in elderly and non-elderly. Presence of food reduces Cmax by approximately 50% without any effect on total exposure in both the genders. All metabolites found in humans have also been found in toxicology species. Human specific metabolite was not observed. This indicates that the animals in the toxicological studies have been exposed to the same metabolites likely to form in clinical setting.

Multiple Ascending Dose Study-

The first cohort of this study had been completed. The first cohort comprised of 16 overweight/obese subjects, out of which there were 11 males and 05 females. Of the 16 subjects dosed, 12 subjects received active treatment and 4 subjects received placebo. Dosing (50mg once daily) took place for 28 days (Days 1–28).

Safety and tolerability parameters (adverse event [AE], electrocardiogram [ECG], vital signs and clinical laboratory test results) data were evaluated at weekly intervals during the study. The safety and tolerability evaluation was done up to 48 hours post dose (relative to the dose administered on Day 28) and of follow-up visits from the first dose level. All these data were reviewed at regular intervals by an independent Data Safety Monitoring Board (DSMB). All the subjects attended for a follow-up visit

8-10 days after the last dose. There has been no safety concern and DSMB has recommended continuation with dosing of subsequent dose level (150mg per day).

No serious adverse events (SAE) and no important medical events occurred during the conduct of the study.

No withdrawal due to adverse events (AE) occurred during the conduct of the study.

One adverse event was observed in one subject. The details are as follows:

Randomisation number	1002
Description	Cough
Start date and time	14/12/10, 2000
End date and time	16/12/10, 1130
Onset after dosing	09 hrs and 58 minutes
Duration	40 hrs and 30 minutes
Severity	Mild
Relationship to study drug	Unrelated
Treatment	Grilinctus cough syrup

Apart from recording vitals at predefined time-points, Ambulatory Blood Pressure and Heart rate was recorded at Baseline, Day 14 and at end of study. No clinically relevant changes in Blood Pressure and Heart rate were observed during the study.

In addition, the effect of TRC150094 on Blood Pressure and Heart rate parameters during exercise was evaluated at baseline and at end of study by Cardiopulmonary Exercise testing. No clinically relevant changes have been observed.

12-lead ECG was recorded in triplicate at each time-point, with each ECG separated by approximately 1 minute at each time point. No clinically relevant changes were observed in ECG during the study and post study follow up. No clinically relevant changes were observed in ECG time intervals : in particular, there was no prolongation of PR interval > 220 msec associated with a greater than 20 msec increase from baseline, prolongation of QRS interval > 120 msec , and of prolongation of QTc (Bazett) interval > 450 msec in subjects.

Cardiac monitoring by ECG was done during exercise while conducting Cardio-pulmonary exercise test. Special attention was given to rhythm of ECG to identify any provocable arrhythmia in these subjects. There has not been any evidence of provocable arrhythmia or ectopics in any of the subjects dosed in first cohort.

The laboratory safety parameters in this study included renal safety markers, bone injury markers, cardiac safety markers and ACTH apart from routine battery of safety tests. No clinically relevant changes in laboratory tests including liver enzymes, renal safety markers, bone injury markers, cardiac safety markers and ACTH, related to intake of TRC150094 were observed throughout the study.

Overall, the safety and tolerability of 50 mg Dose in Multiple Ascending Dose study has been established.

7.2.4 Summary of potential risks and benefits

The ascending doses of TRC150094 ranging from 5 mg to 400 mg, evaluated in the SAD study were found to be safe and tolerable in non-elderly overweight/obese subjects. TRC150094 was also found to be safe and tolerable in elderly cohort. There were no SAEs or withdrawals due to AE in the study. All the adverse events were mild to moderate in nature. Headache was the most common AE observed in both elderly and non-elderly cohorts. Overall, the safety and tolerability up to 400 mg single dose has been established. Based on this data, we have selected 50 mg multiple dose administration (OD for 28 days) as a tentative dose level for this study which is 8 times lower than the dose at which safety in single ascending dose study has been established. Moroever, the Cohort 1 of MAD study has been completed and the safety and tolerability of multiple administrations of 50 mg OD dose level has been established.

7.2.5 Description and justification of route of administration and dosage

The planned dose has been selected based on estimates of safety from pre-clinical studies and safety and tolerability data obtained from SAD study. The study will begin subject to the availability of safety data of 50 mg dose of MAD study. A placebo control has been included in the study design to allow for an unbiased assessment of insulin sensitivity. This part of the study will also be double-blind and randomized to ensure unbiased data.

7.3 Dose Administration

Dosing will start on Day 1. It is planned that each subject will receive once daily dosing (tentatively 50 mg) in the morning for Day 1-28 under fasting conditions with a glass of water. Uniformity in timing of intake of medication will be advised which should be preferably within ± 1 hr of the time of intake of study medication on Day 1. The dosing will not be under direct supervision (except Day 28 dosing); the subjects are required to maintain a diary where they will make entry for the daily dosing. The subjects will be advised not to take any food for at least 1 hr prior to dosing and for 1 hr post-dose. For outpatient days (i.e. on days 1 to 28) drug supply in appropriately labelled bottles will be provided to the subjects. The subjects will be instructed to carefully document the time of drug consumed and time of meals and exercise, in the diary cards provided to them. Subjects will be asked to bring the remaining drugs with the diary cards during intermediary visit to the clinic. The study pharmacist or technician will carry out drug reconciliation and ensure compliance of the subject is satisfactory. Appropriate documentation of the subject specific dispensing process must be maintained. The batch number of each study drug administered to each subject will be recorded in Pharmacy documents.

7.4 Supply, Identification and Storage

The investigational products will be manufactured, handled and stored in accordance with Good Manufacturing Practice (GMP) and used in accordance with this protocol. Torrent Pharmaceuticals Limited (sponsor) will manufacture and provide the TRC150094 and placebo tablets and will ensure that the drug supplies are suitable for human use.

The sponsor will supply Certificates of Analysis for the TRC150094 and placebo tablets, including the batch numbers and expiry dates. The bulk supply will include TRC150094 tablets and Placebo tablets. The labels of the bulk packaging supplied by the sponsor will include the following information:

- Name, address and telephone number of sponsor
- Trial reference code
- Investigator name
- Name of study drug and formulation
- Dose strength
- Route of administration
- Batch number
- Manufacture date
- Expiry date/re-test period
- Storage and reconstitution instructions
- "For Clinical Trial Use only"
- "Keep all medicines out of reach of children"

On receipt, the pharmacy staff at the CRU will check the contents and will complete the appropriate documentation. The study drugs will be kept in a secure, temperature-controlled, restricted-access location and in accordance with applicable regulatory requirements. The study drugs will be stored at a temperature below 25°C, and temperature logs will be maintained. The investigator will ensure that the investigational products are used only in accordance with this protocol.

7.5 Treatment Compliance

Details of study drug administration will be recorded in subject diary. The same will be transcribed to CRF. History of drug compliance will be recorded during the interim safety visit at Day 14±1 and Day 28+2. Drug compliance of at least 90% will be ensured. Hence the allowable limit of missing the dose should not be more than 3

days in total. Discontinuation should not be of more than 2 consecutive days at any point of time.

7.6 Treatment of Overdose

In the event of an overdose of study drug, the subject should be given supportive treatment depending on the symptoms.

7.7 Accountability

The pharmacy staff and the investigator at the CRU will be responsible for drug accountability. Records will be kept of:

- all study drugs delivered to the trial site
- all study drugs dispensed (with batch no.)
- the administration to each subject
- all study drugs remaining and returned/destroyed.

These records will include dates, quantities, batch numbers, expiry dates, and the unique code numbers assigned to the investigational products and trial subjects. Monitor assigned by sponsor (who is not directly associated with conduct of study) will verify the drug accountability records during the study and will perform drug reconciliation at the end of the study. At the end of the study, unused study drugs will be returned to the sponsor or destroyed, as directed by the sponsor (by written authorisation). The return/destruction of all investigational medicinal products will be documented appropriately.

7.8 Dispensing of Investigational Product

The dispensing pharmacist will dispense a quantity of the investigational products sufficient for dose administration well in advance as per the randomization schedule in presence of Quality Assurance (QA) and the remaining Investigational Products will be kept in their original containers. Individual subject supplies for each visit in HDPE bottles will be packed and labelled according to local regulations. For dispensing at Veeda, each subject bottle will contain sixteen tablets (14 for study drug medication once daily and 2 for extra) per visit while for dispensing at AMC, each subject will be provided bottles with 30 tablets/bottle (including 2 reserve tablets). For last dosing on day 28, subject will visit the site on day 27 and subject will be asked to take a tablet (last dose) under supervision.

8. METHODS

8.1 Study parameters/endpoints

8.1.1 Main study parameter/endpoint

To determine safety and efficacy for TRC150094 in increasing hepatic and peripheral insulin sensitivity

8.1.2 Secondary study parameters/endpoints

To determine the effect of TRC150094 on hepatic fat and multiple metabolic parameters

8.2 Randomisation and treatment allocation

8.2.1 Preparation of randomisation code

The randomisation code and sealed individual code-break envelopes will be prepared by the clinical research unit at each site. One set of the individual code-break envelopes will be prepared and provided to the investigator. Each research unit will prepare three randomization lists for statistician, pharmacist and sponsor respectively. The randomisation code will specify whether the subject receives TRC150094 or placebo dosing over the course of the study period. The randomisation code will be in consecutive sequence at the two study centres; hence at one unit it will be 001-020 and other unit it will be 021-040. The randomization list provided to the sponsor nominated individual (who is not directly associated with the study) will be sealed. The seal will be ensured at the end of study. All randomisation information will be secured and kept in a locked storage area, accessible only by authorised personnel. Only subjects who meet all of the inclusion criteria and none of the exclusion criteria are eligible for randomisation. Randomisation will take place on Day 0, the day before dosing. Subjects will be randomized to receive either placebo or TRC 150094 in 1:1 ratio. Each subject will be given a subject number (equivalent to the randomisation number). This number will be a consecutive number from 001 onwards. It will be assigned in the order of the inclusion of subjects at the first dosing.

8.2.2 Breaking the randomisation code

The investigator will have access to one set of sealed code-break envelopes. In the event of an emergency, e.g. a serious adverse event (SAE), the investigator may open the subject’s envelope to determine the study drug administered to the subject. If

possible, the sponsor should be notified before the blind is broken; otherwise, the sponsor will be notified as soon as possible. In addition the IEC (Independent Ethics Committee) will also be notified if the code is broken during the study. The date, time and reason, and the name of the person who opened the envelope, will be recorded in the subject's CRF and on the individual envelope. If the blind is broken, the subject must be withdrawn from the study and replaced.

8.3 Blinding

This will be a double-blind design. The subjects and investigator (and other personnel involved in the study) will be unaware of the study drugs administered to individual subjects.

Personnel in the biometrics department at Academic Medical Centre and Veeda Clinical Research will be blind during the study, except for a nominated individual(s) responsible for preparing the randomisation code and code-break envelopes. The sponsor will remain blind during the study. The placebo tablets will be identical in appearance and taste to the TRC150094 tablets administered at the corresponding dose level, and the same number will be administered. Pharmacy personnel at the study centre who prepare and check the individual unit doses from the bulk packaging will be the only staff at the study centre that is not blind to the study drug. At regular monitoring visits, a study monitor will check the blind is maintained during dosing and during the study, and that all code-break envelopes remain intact. The monitor will remain blind during the study, and steps must be taken during monitoring such that treatment allocation is not revealed.

8.4 Study Procedures

This section describes the Hyperinsulinemic Euglycemic Clamp and Magnetic resonance spectroscopy (MRS) procedures

8.4.1 Hyperinsulinemic Euglycemic Clamp

The clamp procedure will be performed twice on each subject (on Day 0 and Day 28) after an overnight fast. A deviation up to +2 days is acceptable only in those cases that missed doses within the limits given above and has continued planned medication up to day of post-dose investigations. Participants will be admitted to the trial unit and studied in the supine position. During the procedure, the participants are only allowed to drink water. Following a 13 hr fast, a catheter will be inserted in the vein of each

arm. One catheter is used for sampling of arterialised blood using a heated hand box (60°C). The other catheter is used for infusion of [6,6-2H2]-glucose en and [1,1,2,3,3-2H2]-glycerol, glucose 20% and insulin. At t= -2hr, after drawing a blood sample for background enrichment of plasma glucose, a continuous infusion of [6,6-2H2]-glucose (>99% enriched, Cambridge Isotopes, Massachusetts, USA) is started at a rate of 0.11 µmol/kg per min after a priming dose equivalent to 100 min of infusion. After 1 hr, a continuous infusion of [1,1,2,3,3-2H2]-glycerol (>99% ernriched, Cambridge Isotopes, Massachusetts, USA) is started at a rate of 0.11 µmol/kg per min after a priming dose of 1.6 µmol/kg/min. The first 2 infusion hrs are required for equilibration. After 115, 120 and 125 min blood samples are drawn for determination of glucose and glycerol enrichment to calculate basal endogenous glucose production and lipolysis, glucoregulatory hormones and FFA[10]. Subsequently, the hyperinsulinemic clamp will start with a continuous infusion of insulin (Actrapid 100U/ml, Novo Nordisk) for 2hr at a rate of 20mU/m2 body surface area per min. Plasma glucose is measured every 10 min (Biosen C-line plus glucose analyzer; EKF Diagnostics, Barleben/Magdeburg, Germany at AMC, Netherlands; and YSI analyzer at Veeda Clinical Research India) and glucose 20% is infused at a variable rate to maintain plasma glucose at 5.0 mmol/l or 90 mg/dl. [6,6-2H2]-glucose is added to the 20% glucose solution to achieve glucose enrichments of 1% to minimize changes in isotopic enrichment due to changes in the infusion rate of exogenous glucose, and thus to allow for accurate quantification of endogenous glucose production and uptake[10]. During the last 20 min of the hyperinsulinemic clamp, blood samples are drawn at 5 min intervals for determination of glucose and glycerol enrichment, glucoregulatory hormones and FFA. At t= 2.15hr, the infusion of insulin is increased to a rate of 60mU/m2 body surface area per min. Plasma glucose is measured again every 10 min and enriched glucose 20% is infused at a variable rate to maintain plasma glucose at 5.0 mmol/l or 90 mg/dl. During the last 20 min of this insulin infusion step, blood samples are drawn at 5 min intervals for determination of glucose and glycerol enrichment, glucoregulatory hormones and FFA. Thereafter insulin infusion will be discontinued and participants will be offered a carbohydrate rich meal. The glucose infusion rate will be doubled. Plasma glucose levels will be measured every 10 minutes for the first hour. Thereafter blood glucose measurement will be done at 20 minute intervals or more frequently (at the discretion of the

investigator). Every time plasma glucose levels are > 7 mmol/l the glucose infusion rate will be halved until less than 5 ml/hr. Four weeks later, the second clamp will be performed with the same methodology.

Name and description of investigational product(s)

- [6,6-2H2] labelled glucose, Cambridge isotopes, Cambridge, Massachusetts, USA
- [1,1,2,3,3-2H2] labelled glycerol, Cambridge isotopes, Cambridge, Massachusetts, USA
- Actrapid, Novo Nordisk
- Glucose 20%

[6,6-2H2] labelled glucose and [1,1,2,3,3-2H2] labelled glycerol will be involved at the pharmacy at the study centre and will be prepared by the investigators.

Endogenous glucose production (EGP) and peripheral glucose uptake (Rd) are calculated using the modified form of the Steele equations.[28] Lipolysis (glycerol turnover) will be calculated by using formulas for steady state kinetics adapted for stable isotopes.

8.4.2 Quantification of Hepatic Fat.

Hepatic will be measured by ^{1}H MRS on Day 0 and Day 28. A deviation ± 2 days is acceptable. The analysis and interpretation of MRS data will be done by core lab of Academic Medical Centre at Amsterdam, The Netherlands. During the measurements, the subjects will lie down inside the magnet of a clinical magnetic resonance spectrometer with a body array surface coil positioned around the abdomen. For the quantification of liver fat, localized single voxel proton MR spectra from the liver will be obtained without water suppression using long repetition time and short echo time to minimize relaxation effects on signal intensity. The MRS measurement will be carried out without breath holding Spectral signal intensities of water and methylene groups originating from the lipids will be fitted to determine the percentage of intracellular triglycerides of the hepatocytes[14].

8.4.3 Silent Information Regulator T (SIRT) expression study:

Modulation of SIRT expression will be monitored for Indian cohorts only. For this purpose, 10ml of heparinised blood will be collected. Peripheral blood mononuclear cells (PBMNCs) will be isolated using HISTOPAQUE within 2 hrs of blood collection. Isolated PBMNCs will be stored at -80ºC. Total RNA will be isolated from PBMNCs and SIRT gene expression will be monitored employing specific probes by real-time PCR. Modulation of SIRT expression will be monitored for each volunteer at Day 0, Day 14 and Day 28 of the study. A deviation of ±1 day is acceptable. Analysis of samples for SIRT expression will be performed at Cell and Molecular Biology Lab, Torrent, India.

Total 30 ml heparinised blood per volunteer will be required.

8.4.4 Measurement of Sagittal Abdominal diameter

Sagittal abdominal diameter will be measured in the supine position with bent knees on a firm examination table. The measurement will be done to the nearest 0.1 cm after a normal expiration and without clothes in the measurement area. Sagittal abdominal diameter will be measured at the level of iliac crest (L4–5 using a sliding-beam caliper, as the distance between the examination table up to the horizontal level, allowing the caliper arm to touch the abdomen slightly but without compression[29].

8.5 Study Visits

Screening

Subjects will attend the CRU for a screening visit up to 28 days before their Baseline investigation (Day 0). Prior to attending for a screening visit, subjects will be asked to comply with certain exercise and dietary restrictions, as specified in Section 6.7. Informed consent must be obtained at this visit before any study procedures are performed. Further details regarding informed consent are provided in Section 11.2. A screening log will be kept to record subjects who sign the informed consent form and who are screened. For those subjects who are screen failures, a reason will be documented. The following assessments/information will be performed/ recorded

- date of birth and age
- race

- height and body weight (measured while wearing indoor clothing and no shoes)
- waist circumference
- body mass index (BMI), calculated as weight (kg)/height (m)2
- smoking status / intake of tobacco in any other form (including current and historical use of tobacco, and ability to stop smoking / tobacco intake in any other form, for study periods)
- medical history (any significant conditions or diseases that stopped at or prior to screening)
- pre-existing (concurrent) conditions (those present at the screening visit)
- prior and ongoing medication (medications taken up to 4 weeks prior to first dose)
- full physical examination
- vital signs (supine and standing) and body temperature
- 12-lead ECG
- chest X-ray (to exclude active tuberculosis, in India only)
- clinical laboratory tests (blood sample for haematology and biochemistry tests; and urine sample for urinalysis)
- lipid Parameters
- viral serology (hepatitis B surface antigen [HBsAg], hepatitis C antibody, human immunodeficiency virus [HIV I and HIV II])
- alcohol breath test
- urinary drugs of abuse screen (amphetamines, barbiturates, benzodiazepines, cannabinoids, cocaine, opiates and methadone).

Those subjects who are eligible for the study, based on the inclusion and exclusion criteria, will be invited to take part in the study. Lifestyle instructions as mentioned in Section 6.7 will be given.

Day 0

Those subjects who meet all inclusion and none of the exclusion criteria and who have given their informed consent will be asked to come to the CRU for baseline investigations on Day 0. On Day 0 subjects will visit the study centre early in the morning after a 13 hr fast. If it is not possible for a subject to arrive at the study centre this early in the morning, the subject is offered to stay overnight. The subjects may be asked to visit the CRU on Day-1 for some baseline investigations (at the discretion of Principal Investigator). Randomisation will take place on Day 0.

At Day 0 the following assessments will be performed

- physical examination
- vital signs
- 12-lead ECG
- height and body weight
- waist circumference
- Sagittal abdominal diameter
- clinical laboratory tests (Haematology, Biochemistry, urine analysis)
- Blood Sample for SIRT expression (see Appendix B)
- lipid Parameters
- hepatic MRS – See Section 8.4.2 (a deviation of ± 2 days is acceptable for MRS
- compliance to drug and life style instructions
- AEs and change in concomitant medications will be reported
- hyperinsulinemic euglycemic clamp, the clamp procedure is described in Section 8.4.1

Treatment Period Day 1-28

Each subject will receive once daily dosing in the morning for Day 1-28 under fasting conditions (see Section 7.5)

Day 14

On Day 14, subjects will visit the CRU after an overnight fast. A deviation ± 1 day is acceptable. The following assessments will be performed:

- vital signs
- height and body weight
- waist circumference
- Safety clinical laboratory tests (Haematology, Biochemistry, urine analysis)
- Blood Sample for SIRT expression (see Appendix B)
- lipid Parameters
- 12-lead ECG
- AE check and change in concomitant medications will be recorded
- compliance to drug and Life style instructions

Day 28

On Day 28 subjects will visit the study centre early in the morning after a 13 hr fast. A deviation up to +2 days is acceptable only in those cases that missed doses within the limits given above and has continued planned medication up to day of post-dose investigations. If it is not possible for a subject to arrive at the study centre this early in the morning, the subject is offered to stay overnight. The subjects may be asked to visit the CRU on Day 27 for some end-of-treatment investigations (at the discretion of Principal Investigator).

At Day 28 the following assessments will be performed.

- physical examination
- vital signs
- 12-lead ECG
- height and body weight
- waist circumference
- Sagittal abdominal diameter
- clinical laboratory tests (Haematology, Biochemistry, urine analysis)
- Blood Sample for SIRT expression (see Appendix B)
- lipid Parameters

- hepatic MRS– See Section 8.4.2 (a deviation of ± 2 days is acceptable for MRS)
- AEs and change in concomitant medications will be reported
- hyperinsulinemic euglycemic clamp, the clamp procedure is described in Section 8.4.1
- Compliance to drug and Life style instructions
- Discontinuation of study drug

At follow up visit (Day 35), subjects will visit the study centre after an overnight fast.

Follow up visit

Follow-up visit is planned at Day 35. A deviation of + 3 days is acceptable.

At Day 35 the following assessments will be performed:

- Physical examination
- Vital signs
- Height and body weight
- Waist circumference
- Safety clinical laboratory tests (Haematology, Biochemistry, urinanalysis)
- Lipid Parameters
- 12-lead ECG
- AEs and change in concomitant medications will be reported
- Restart of metformin treatment (if applicable)

8.5.1 . Clinical laboratory tests

A blood sample will be taken for measurement of haematology and biochemistry parameters, and a urine sample will be taken for urinalysis at the following time points:

- screening visit
- Day 0
- Day 14
- Day 28

- Day 35

The Laboratory parameters and time-points are specified in (Appendix A) and (Appendix B).

8.5.2 Vital signs

Body temperature and supine vital signs (blood pressure and heart rate) will be measured on the non-dominant arm after 5 minutes of supine rest on the Day of screening visit, Day 0, Day 14±1 and Day 28 (t = -2hr) and at follow up (See Appendix B). Standing vital signs (blood pressure and heart rate) will also be measured at the screening visit. Standing vital signs will be measured after the supine values have been recorded and after the subject has been standing for 2 minutes. These measurements will be used to calculate the postural drop in blood pressure. Vital sign measurements will be performed within ±10 minutes of the scheduled time points

8.5.3 12-Lead ECGs

12-Lead ECGs will be recorded after 5 minutes supine rest on the Day of screening visit, Day 0, Day 14±1, Day 28 and at follow up (See Appendix B). The 12-lead ECGs will be recorded in triplicate at each time point, with each ECG separated by approximately 1 minute at each time point.

8.5.4 Physical examinations

A full physical examination will be performed on the day of screening visit. Abbreviated Physical examination will be done on Day 0, Day 28 and Follow-up.

8.5.5 Chest X ray

Chest X ray will be performed only in India and on the day of screening to exclude pulmonary TB-infection.

8.5.6 Laboratory investigations and bioanalysis of blood samples

The sampling schedule for laboratory assessments are as mentioned in Appendix B. The Safety laboratory investigations will be done at the local lab of the respective study centre. The analysis of metabolic biomarkers (mentioned in Appendix A and B), endocrinology assessments of all 40 Subjects will be done at Academic Medical Centre, The Netherlands. Also, bioanalysis of blood samples for the determination of

glucose and glycerol enrichment of all 40 Subjects will be done at Advanced Laboratory of Endocrinology, AMC, Meibergdreef 9, room F2-131.3, 1100 DD Amsterdam, The Netherlands. Blood samples collected from Study subjects at Veeda Clinical Research Ahmedabad for evaluation of the parameters mentioned above will be sent to AMC, The Netherlands.

Blood sampling

Blood sampling will be done for glucose monitoring (for maintaining Euglycemia) at every 10 minutes. A window of ±1 minute will be allowed. Blood samples will be taken at the following time points relative to the start of continuous glucose infusion (taken as t=0): T= -2:00, -0:05, -0:00, 0:05, 1:50, 1:55, 2:00, 2:05, 2:10, 4:00, 4:05, 4:10, 4:15, 4:20 (14 samples during one clamp procedure).

The actual time of blood sampling will be recorded in the CRF. At each time point, the blood samples will be collected into the appropriate polypropylene tube. The tubes will be inverted gently 8 to 10 times before processing. The tubes will be kept in an ice bath before centrifugation. Within 30 minutes of blood sample collection, the samples will be centrifuged at approximately 3000 g for 10 minutes at 4ºC. The plasma obtained from each sample will then be transferred into four labeled polypropylene tubes and frozen immediately. The tubes will be stored at a temperature below −70ºC prior to shipment. One set of samples will be sent to the Advanced Laboratory of Endocrinology, AMC the Netherlands for bioanalysis of each d_2-glucose and d_5-glycerol, insulin, cortisol, glucagon, catecholamines, other biomarkers and as duplicate and back-up. All safety laboratory investigations will be done at the local lab of the respective study centre.

Shipment

The plasma samples for bioanalysis will be shipped to the following bioanalytical facility on dry ice, with a portable data logger (sample shipment log):

Laboratory of Special Endocrinology, AMC

Meibergdreef 9, room F2-131.3

1100 DD Amsterdam, The Netherlands

The samples will be checked by the bioanalytical facilities as soon as possible after they are received, and receipt will be documented.

8.5.7 Bioanalysis

Plasma samples will be analysed using a validated GCMS method.

8.5.8 Sample retention / destruction

The plasma samples will be retained by the bioanalytical facilities for 1 month after the final study report has been signed off. The samples will be destroyed/returned back to sponsor after obtaining written consent from the sponsor for the same. Duplicate Samples will be sent to the bioanalytical facility after the confirmation of receipt of first set is provided to the study centre.

8.6 Appropriateness of Measurements

All planned assessments are standard measurements for this type of study and are considered appropriate.

8.7 Total Volume of Blood

Table 1: Summary of Blood Volumes for Each Subject

Requirement	Number and Volume of Samples	Total
Clinical laboratory tests		
Hematology, endocrinology	20 x 4.5 ml	90
Haemostasis	5 x 2.7 ml	13.5
Biochemistry	5 x 4.5 ml	22.5
Viral Serology	1 x 10 ml	10
SIRT(for subjects at Veeda CR only)	3 x10 ml	30
Clamp Day (including baseline measurements, reserve samples and glucose during clamp)	2 x (125.1ml+ 20x 0.5ml (glucose))	270.2
**Total**<br>**For subject at AMC**<br>**For subject at Veeda CR**		 **406.2ml**<br>**436.2 ml**

8.8 Withdrawal Criteria

Subjects may decide to withdraw from the study at any time without prejudice to their further medical care. Although a subject is not obliged to give his reason(s) for withdrawing prematurely from a trial, the investigator should make a reasonable effort to ascertain the reason(s) while fully respecting the subject's rights. The investigator may withdraw a subject for any of the following reasons:

- adverse event: if subject is unwilling to continue because of an AE or if continued participation of the subject would be an unnecessary risk to the subject's health, in the opinion of the investigator
- non-compliance
- protocol deviation
- lost to follow-up
- study blind is broken
- sponsor request
- following review of safety/tolerability data
- other.

8.9 Withdrawal Procedures

If a subject withdraws from the study, the primary reason for withdrawal must be recorded in the CRF. The procedures scheduled for early withdrawal should be performed if possible, as well as any other additional procedures requested by the investigator for safety purposes. The last date of study drug administration must be documented. Appropriate follow-up of withdrawn subjects will be performed, as required. Attempts to contact a subject who withdraws from a study must be documented.

8.10 Replacement of Dropout/Withdrawals

Replacement of dropout/withdrawals will be done by Pharmacy personnel at the CRU, who is unblind to the study drug. Pharmacist at the CRU will manage any dropout/withdrawal by providing different subject ID and randomisation code to the reserve subject but will receive same drug (active treatment/placebo) as allotted to the replaced subject. For example, if subject 002 is withdrawn, then the first replacement

will be allocated with randomisation code A002, if second replacement of same subject happen then B002, for third replacement C002.

8.11 Follow up of subjects withdrawn from treatment

Should a subject request or decide to withdraw from the study, all efforts will be made to complete and report the observations as thoroughly as possible up to the date of withdrawal, and for subjects who are withdrawn, the follow-up assessments should be performed within 8-10 days after the last drug administration.

8.12 Study Termination

The sponsor, investigator or Institutional Review Board (IRB)/Independent Ethics Committee (IEC) may terminate the study at any time if any of the following criteria are met.

- There is new information about the study drug that indicates the risk/benefit profile is no longer acceptable for the study to continue.
- There is a significant deviation from the protocol or violation of Good Clinical Practice (GCP) that compromises the study results, subject safety or ability to address the study objectives.

  If a trial is prematurely terminated or suspended for any reason, the investigator should promptly inform the trial subjects and should assure appropriate therapy and follow-up. The sponsor, IRB/IEC and regulatory authority (ies) should be notified

9. SAFETY REPORTING

9.1 Safety and tolerability assessments and reporting

9.1.1 Safety Monitoring

In this study with small sample size and short duration, the independent physician will perform safety control by reviewing clinical safety parameters throughout the conduct of the study. Clinical safety parameters will include laboratory analysis, urine analysis and ECG

9.1.2 Section 10 WMO event

In accordance to section 10, subsection 1, of the WMO, the investigator will inform the subjects and the reviewing accredited IEC if anything occurs, on the basis of

which it appears that the disadvantages of participation may be significantly greater than was foreseen in the research proposal. The study will be suspended pending further review by the accredited IEC, except insofar as suspension would jeopardise the subjects' health. The investigator will take care that all subjects are kept informed.

9.1.3 Adverse events and serious adverse events

Adverse events are defined as any undesirable experience occurring to a subject during a clinical trial, whether or not considered related to the investigational drug. All adverse events reported spontaneously by the subject or observed by the investigator or his staff will be recorded. A serious adverse event is any untoward medical occurrence or effect that at any dose

1. results in death;
2. is life threatening (at the time of the event);
3. requires hospitalisation or prolongation of existing inpatients' hospitalisation;
4. results in persistent or significant disability or incapacity;
5. is a congenital anomaly or birth defect;
6. is a new event of the trial likely to affect the safety of the subjects, such as an unexpected outcome of an adverse reaction, lack of efficacy of an IMP used for the treatment of a life threatening disease, major safety finding from a newly completed animal study, etc.

All SAEs will be reported to the accredited IEC that approved the protocol, according to the requirements of that IEC.

9.1.4 Reporting of adverse event

For each subject, all AEs will be collected from the time of first dose administration through to the post-study follow-up visit. On arrival at the trial unit, at regular intervals during study, and at follow up, each subject will be asked a non-leading question such as "How have you been feeling since last asked?" Any AEs reported in response to questioning, as well as AEs reported spontaneously and occurring at any other time after dosing, will be recorded in the subject's CRF. For each AE, the following information will be recorded:

- AE description
- start and stop dates and times

- single episode or intermittent
- severity: mild (easily tolerated), moderate (interferes with daily activities) or
- severe (prevents normal daily activities)
- relationship to study drug (definite, probable, possible, unlikely or not related)*
- outcome (resolved, resolved with sequelae, or ongoing)
- whether serious or not.

* Definitions for the relationship to study drug assessment, to be made by the investigator, are as follows:

Definite: The AE follows a reasonable temporal sequence from administration of the drug, abates on withdrawal of study drug and re-appears upon re-administration (rechallenge).

Probable: The AE follows a reasonable temporal sequence from the administration of the drug, abates on withdrawal of study drug and cannot be reasonably explained by the known characteristics of the subject's clinical state or other factors, such as concomitant medication.

Possible: The AE follows a reasonable temporal sequence from the administration of the drug but could have been produced by the subject's clinical state or other factors, such as study procedures or concomitant medication.

Unlikely: The temporal association between the AE and the drug is such that the drug is unlikely to have any reasonable association with the AE.

Not related: The AE does not follow a reasonable temporal sequence from the administration of the drug or the AE can be reasonably explained by other factors, such as concomitant medication.

9.1.5 Procedures for reporting SAEs

All SAEs should be reported immediately to the sponsor. The immediate reports should be followed promptly by detailed, written reports. The immediate and follow-up reports should identify a subject by his initials and the subject number, and not by personal information such as name or address. The investigator should also comply

with the applicable regulatory requirement(s) related to the reporting of unexpected serious adverse drug reactions to the regulatory authority(ies) and the IRB/IEC.

9.1.6 Emergency procedures

The subject should be given supportive treatment depending on the symptoms.

9.1.7 Suspected unexpected serious adverse reactions (SUSAR)

Adverse reactions are all untoward and unintended responses to an investigational product related to any dose administered. Unexpected adverse reactions are adverse reactions, of which the nature, or severity, is not consistent with the applicable product information (e.g. Investigator's Brochure for an unapproved IMP or Summary of Product Characteristics (SPC) for an authorised medicinal product). The sponsor will report expedited the following SUSARs to the IEC: SUSARs that have arisen in the clinical trial that was assessed by the IEC; SUSARs that have arisen in other clinical trial of the same sponsor and with the same medicinal product, and that could have consequences for the safety of the subjects involved in the clinical trial that was assessed by the IEC. The remaining SUSARs will be recorded in an overview list (line-listing) that will be submitted once every half year to the IEC. This line-listing provides an overview of all SUSARs from the study medicine, accompanied by a brief report highlighting the main points of concern. The sponsor will report expedited all SUSARs to the competent authority, the Medicine Evaluation Board and the competent authorities in other Member States. The expedited reporting will occur not later than 15 days after the sponsor has first knowledge of the adverse reactions. For fatal or life threatening cases the term will be maximal 7 days for a preliminary report with another 8 days for completion of the report.

9.1.8 Annual safety report

In addition to the expedited reporting of SUSARs, the sponsor will submit, once a year throughout the clinical trial, a safety report to the accredited IEC's, competent authority, Medicine Evaluation Board and competent authorities of the concerned Member States. This safety report consists of:

- a list of all suspected (unexpected or expected) serious adverse reactions, along with an aggregated summary table of all reported serious adverse reactions, ordered by organ system, per study;

- a report concerning the safety of the subjects, consisting of a complete safety analysis and an evaluation of the balance between the efficacy and the harmfulness of the medicine under investigation.

9.1.9 Follow-up of adverse events

All adverse events will be followed until they have abated, or until a stable situation has been reached. Depending on the event, follow up may require additional tests or medical procedures as indicated, and/or referral to the general physician or a medical specialist.

10. DATA MANAGEMENT AND STATISTICAL ANALYSIS

10.1 Sample Size Calculation

It is intended that 40 subjects (20 subjects in India and other 20 in Amsterdam, Netherlands) will complete the study. No formal sample size calculation has been performed. We calculated that a sample size of 20 in each group will have 80% power to detect an absolute difference in Rd of at least 15 µmol/kg·min (measure for peripheral insulin sensitivity), before and after treatment, using a Wilcoxon (Mann-Whitney) rank-sum test with a 0.05 two-sided significance level and assuming that the common standard deviation is 15.

10.2 Data Handling

Data obtained in this study will be collected in eCRF prepared by the CRU of the Academic Medical Centre, Amsterdam. The eCRF will be completed and monitored in accordance with the principles of GCP. Source data is defined as all information in original records and certified copies of original records of clinical findings, observations, or other activities in a clinical trial necessary for the reconstruction and evaluation of the trial. Source documents are original documents, data and records, such as laboratory printouts, 12-lead ECG reports, dispensing records, and subject files. Data recorded directly into the eCRFs will be considered as source. Full details of procedures for data handling will be documented in the Data Management Plan. AEs, medical history, and pre-existing (concurrent) conditions will be coded using the Medical Dictionary for Regulatory Activities (MedDRA). Drugs will be coded using the World Health Organization (WHO) Drug Dictionary.

10.3 Statistical Analysis

10.3.1 Descriptive statistics

Demographic and baseline characteristics will be summarized using descriptive statistics for each group.

10.3.2 Univariate analysis

Because of the small sample size non parametric tests will be used to compare results between the two treatment groups before and after treatment. Actual and percentage change from baseline will be presented. All results are expressed as means and standard deviations when the results are normally distributed or as median and range

when they are not normally distributed. The data will be analyzed using SPSS, version 16.

10.4 Safety and Tolerability Analysis

Safety and tolerability data will be summarised using the Safety Set, broken down by dose level and for subjects on active treatment and on placebo. All AEs will be coded using MedDRA. Data will be summarised using preferred term and primary system organ class. Only treatment-emergent AEs, being events with an onset at or after the first administration of study drug, will be presented in summary tables. Where changes in severity are recorded in the CRF, the most severe incidence of the AE will be reported in the tables. Rates will be calculated as the proportion of the number of subjects with at least one AE related to the number of subjects treated. Frequency tables indicating seriousness, severity, drug relation and the number of subjects will be presented. Summary tables will present descriptive statistics for biochemistry and haematology parameters, corrected QT (QTc) interval, diastolic and systolic blood pressure and heart rate measurements. Frequency tables will be presented for physical examination findings and urinalysis. Qualitative changes in laboratory values will be presented by shift tables. No significance testing will be performed on safety and tolerability data.

11. ETHICAL AND REGULATORY CONSIDERATIONS

11.1 Regulation statement

This study will be conducted in accordance with the protocol, the ethical principles that have their origin in the Declaration of Helsinki, and that are consistent with the principles of the International Conference on Harmonisation (ICH) (Step 5) 'Guidance on Good Clinical Practice', ICMR guidelines, and with procedures oriented to Good Laboratory Practice, and the applicable regulatory requirement(s).

11.2 Recruitment and consent

Subjects will be recruited from the outpatient’s clinic of AMC or from local advertisement or from pre-existing database of the study centre. A study-specific subject information sheet and consent form for each part of the study will be prepared in accordance with the principles of GCP, the Declaration of Helsinki and all

applicable laws and regulations. The information sheet will explain clearly the nature of the study and study drug, including the study objectives, the potential risks and benefits, and the procedures involved for the subject if he was to take part. For each individual volunteer, the investigator or designee will provide a detailed explanation of the study, both verbally and with the use of the written subject information sheet. If the subject decides to participate in the study, the informed consent form must be signed and dated by the subject and the investigator (or designee who conducted the informed consent discussion). The original signed informed consent form must be stored in the trial master file at the trial unit. The date that informed consent was provided must be recorded in the subject's CRF. A copy of the signed informed consent form and the information sheet must be given to the subject. The study-specific informed consent form must be signed before any protocol-related procedures are performed. The subject information sheet should be revised whenever important new information becomes available that may be relevant to the subject's consent. Any revised written information and consent form should receive the IEC's approval prior to use.

11.3 Benefits and risks assessment, group relatedness

This study does not have specific advantages for the study subjects. The results of this study may help researchers learn whether TRC150094 may be beneficial for the treatment of male subjects with the metabolic syndrome. The most important known adverse event of TRC150094 is headache. [6,6-2H2] glucose is glucose labelled with a stable isotope of hydrogen, which behaves as the natural substrate and has no side effects. [1,1,2,3,3-2H2] labelled glycerol is glycerol labelled with 5 stable isotope of hydrogen, which behaves as the natural substrate and has no side effects. Actrapid is fast acting insulin, a hormone that could induce hypoglycemia. However, it is not in the scope of this protocol to allow hypoglycemia to occur, since plasma glucose concentration will be fixed at 5 mmol/l by a variable infusion of glucose 20% guided by frequent bedside glucose measurements An overview of the blood samples taken during the clamp is provided in Appendix B. The total volume of blood sample is 361.2.ml for AMC subjects and 391.2 ml for Indian subjects. This amount is not considered to be of negative influence to the subject's health. MR- spectroscopy of the liver will be made which takes about 30 minutes. This spectroscopy is not considered to be potentially harmful to subjects. An X-thorax will be performed

during the screening visit, to exclude TB-infection, in patients in India only. The radiation hazard of the X-thorax is 0.02 milliSievert per X-ray. For comparison: background radiation is 2 milliSievert per year for every person.

Incentives: In the Netherlands patients will receive 100 euro for each study visit (not screening), this includes reimbursement for travel costs.

11.4 Compensation for study related illness/injury

Subject will be treated and/or compensated for any study-related illness/injury pursuant to the information provided in the Compensation for Injury section of the Informed Consent. Compensation to study subjects will be as per the local regulatory practices. Every subject is insured in accordance with the local laws against damage to health which might occur during the conduct of study and the material damage which occur in connection thereto. The subject insurance and travel insurance (if appropriate) is taken by the Sponsor.

12. ADMINISTRATIVE ASPECTS AND PUBLICATION

12.1 Handling and storage of data and documents

The investigator should maintain a list of appropriately qualified persons to whom he/she has delegated trial duties. All persons authorized to make entries and/or corrections on eCRFs will be included on the Authority Form. The investigator must ensure that the subject's confidentiality is maintained. On the eCRFs or other documents subjects should be identified by subject ID and randomization number only. The Investigator is obligated to inform and obtain the consent of the subject to permit named representatives to have access to his/her study-related records without violating the confidentiality of the subject. Source documents are the original documents, data, and records from which the subject's eCRF data are obtained. These include but are not limited to hospital records, clinical and office charts, laboratory and pharmacy records, diaries, microfiches, radiographs, and correspondence.

The Investigator and study staff are responsible for maintaining a comprehensive and centralized filing system of all study-related (essential) documentation, suitable for inspection at any time by representatives from the Sponsor or designee, and/or applicable regulatory authorities. Elements should include:

• Subject files containing informed consents, and supporting copies of source documentation

• Study files containing the protocol with all amendments, Investigator's Brochure, copies of pre-study documentation, and all correspondence to and from the IEC as well as to and from the Sponsor or designee.

• Study files containing documentation of study drug receipt, accountability, return, and all drug related correspondence In addition, all source documents supporting entries in the eCRFs must be maintained and be readily available. No study document should be destroyed without prior written agreement between the Sponsor or designee and the Investigator. Should the Investigator wish to assign the study records to another party or move them to another location, he/she must notify the Sponsor or designee in writing of the new responsible person and/or the new location. The Sponsor representative or designee and regulatory authority inspectors are responsible for contacting and visiting the Investigator for the purpose of inspecting the facilities and, upon request, inspecting the various records of the trial (for example, eCRFs and other pertinent data) provided that subject confidentiality is respected. The Sponsor representative or designee is responsible for inspecting the eCRFs at regular intervals throughout the study to verify adherence to the protocol; completeness, accuracy, and consistency of the data; and adherence to local regulations on the conduct of clinical research. The monitor should have access to subject medical records and other study-related records needed to verify the entries on the eCRFs. In accordance with ICH GCP and the Sponsor's audit plans, this study may be selected for audit by representatives from the Sponsor's Clinical and Quality Assurance Department (or designees). Inspection of study center facilities (e.g., pharmacy, drug storage areas, laboratories) and review of study-related records will occur to evaluate the trial conduct and compliance with the protocol, ICH GCP, and applicable regulatory requirements.

12.2 Amendments

Amendments are changes made to the research after a favourable opinion by the accredited IEC has been given. All amendments will be notified to the IEC that gave a favourable opinion. A 'substantial amendment' is defined as an amendment to the terms of the IEC application, or to the protocol or any other supporting documentation, that is likely to affect to a significant degree:

- the safety or physical or mental integrity of the subjects of the trial;
- the scientific value of the trial;
- the conduct or management of the trial; or
- the quality or safety of any intervention used in the trial.

All substantial amendments will be notified to the IEC and to the competent authority.

Non-substantial amendments will not be notified to the accredited IEC and the competent authority, but will be recorded and filed by the sponsor.

12.3 Annual progress report

The sponsor/investigator will submit a summary of the progress of the trial to the accredited IEC once a year. Information will be provided on the date of inclusion of the first subject, numbers of subjects included and numbers of subjects that have completed the trial, serious adverse events/ serious adverse reactions, other problems, and amendments.

12.4 End of study report

The sponsor will notify the accredited IEC and the competent authority of the end of the study within a period of 90 days. The end of the study is defined as the subject's last visit. In case the study is ended prematurely, the sponsor will notify the accredited IEC and the competent authority within 15 days, including the reasons for the premature termination. Within one year after the end of the study, the investigator/sponsor will submit a final study report with the results of the study, including any publications/abstracts of the study, to the accredited IEC and the CompetentAuthority.

12.5 Public disclosure and publication policy

Upon completion of the trial (or early termination), the investigator should provide the IEC with a summary of the trial's outcome, and the regulatory authority(ies) with any reports required. A clinical study report will be prepared in accordance with the ICH E3 guideline: Note for guidance on structure and content of clinical study reports (CPMP/ICH/137/95). The detailed obligations regarding the publication of data or information from this study will be set out in a separate agreement between the investigator and sponsor. The identity of individual subjects will not be revealed in any reports or publications.

13. QUALITY CONTROL AND QUALITY ASSURANCE

The following activities will be undertaken to ensure the quality of trial-related activities:

- adherence to the trial site standard operating procedures to maintain accurate and consistent practices and procedures
- conduct of a site initiation visit to ensure the investigator and all personnel involved in the trial understand the protocol, including the study procedures and their responsibilities
- completion of eCRF in accordance with GCP requirements to ensure accurate and reliable data
- periodic monitoring to ensure the trial data are accurate, complete and verifiable from source documents, and that the protocol is being followed
- data entry of eCRF data and data management of all trial data will be performed in accordance with GCP requirements.

The trial site may be subject to quality assurance audits by the sponsor or its designee. If an audit is undertaken, the site will be contacted in advance to arrange an auditing visit.

APPENDIX A: Clinical Laboratory Tests

Haematology<br>Red blood cells<br>Total Leukocytes count (cell count)<br>Differential WBC count (absolute and %) including neutrophils, eosinophils, basophils, monocytes, lymphocytes<br>Haemoglobin<br>Platelets (cell count)<br>Packed cell volume<br>ESR	Biochemistry<br>Total Protein<br>Albumin<br>Urea (BUN)<br>Creatinine<br>Fasting Blood Glucose & Insulin<br>Electrolytes: Serum Na, K, HCO3,Ca, P, Cl^-<br>Liver Function Tests: ALT, AST, Bilirubin (Total and Direct)<br>LDH, GGT
Haemostasis<br>Prothrombin time,<br>aPTT (Activated<br>Partial thromboplastin time),<br>Fibrinogen.	Metabolic markers:<br>FFA, Leptin, TNF-alpha, IL-6, IL-1beta, IL-10, MCP-1, Serum Glucagon, Serum Epinephrine, Resistin, Adiponectin, Apolipoproteins, CRP, Other metabolic markers (Refer Appendix B).
Endocrinology<br>Thyroid Profile (T3, T4, fT4, TSH)	
Urinalysis<br>pH, specific gravity, ketones, protein,glucose, urobilinogen, bilirubin, nitrite, erythrocytes, leukocytes	Lipid Parameters<br>Total cholesterol, TG,<br>LDL – Cholesterol, VLDL, HDL
Viral serology<br>HBsAg, hepatitis C antibody, HIV I, HIV II	Drugs of abuse<br>amphetamines, barbiturates, benzodiazepines, cannabinoids, cocaine, opiates, methadone<br>Alcohol breath test

Time points for the clinical laboratory tests are mentioned in Appendix B.

APPENDIX B: Study visits.

1	S	Day 0														Day 14	Day 28	Day 35
		0 min	115 min	120 min	125 min	During clamps												
						Last 20 mins (at 5 min intervals)					Last 20 mins (at 5 min intervals)							
**Invest**	**T**	**-2:00**	**-0:05**	**0:00**	**0:05**	**1:50**	**1:55**	**2:00**	**2:05**	**2:10**	**4:00**	**4:05**	**4:10**	**4:15**	**4:20**			
Informed consent	×																	
Demographics, smoking status	×																	
Height, weight, BMI, Waist Circ	×	×														×	×	×
Sagittal abdominal diameter		×															×	
Medical history; pre-existing conditions	×																	
Prior/concomitant medication check	×																	
Physical examination	×	×															×	×
Vital signs	×	×														×	×	×
Body temperature	×																	
12-Lead ECG	×	×														×	×	×
Chest X ray (In India only)	×																	
Hematology	×	×														×	×	×
Safety Biochemistry	×	×														×	×	×
Serology	×																	
Lipid profile	×	×														×	×	×
Urine Analysis	×	×														×	×	×

**2**	**S**	**Day 0**														**Day 14**	**Day 28**	**Day 35**
		0 min	115 min	120 min	125 min	During clamps												
						Last 20 mins (at 5 min intervals)					Last 20 mins (at 5 min intervals)							
**Invest**	**T**	**-2:00**	**-0:05**	**0:00**	**0:05**	**1:50**	**1:55**	**2:00**	**2:05**	**2:10**	**4:00**	**4:05**	**4:10**	**4:15**	**4:20**	**Not applicable**	**At day 28 the same scheme as day 0 will be followed**	**Not applicable**
D2 glucose		×	×	×	×	×	×	×	×	×	×	×	×	×	×			
D5 glycerol		×	×	×	×	×	×	×	×	×	×	×	×	×	×			
Serum insulin	×		×	×	×		×	×	×			×	×	×				
Serum glucagon				×				×					×					
Catecholamines				×				×					×					
cortisol				×				×					×					
FFA				×				×					×					
Leptin				×				×					×					
TNF Alpha				×				×					×					
IL-6				×				×					×					
IL-1beta				×				×					×					
IL-10				×				×					×					
MCP-1				×				×					×					
Other Metabolic markers (such as Plasma SCD-1 activity, plasma 3-OHB, osteopontin)#				×				×					×					

**3**	**S**	**Day 0**														**Day 14**	**Day 28**	**Day 35**
		0 min	115 min	120 min	125 min	During clamps												
						Last 20 mins (at 5 min intervals)					Last 20 mins (at 5 min intervals)							
**Invest**	**T**	**-2:00**	**-0:05**	**0:00**	**0:05**	**1:50**	**1:55**	**2:00**	**2:05**	**2:10**	**4:00**	**4:05**	**4:10**	**4:15**	**4:20**	**Not applicable**	**Same scheme as day 0**	**Not applicable**
Resistin				×				×					×					
Adiponectin(Total and HMW)				×				×					×					
Apo A1				×				×					×					
Apo B				×				×					×					
CRP				×				×					×					
SIRT expression (For Indian Cohorts only)		×														×	×	×
Drugs of abuse (urine)	×																	
Alcohol breath test	×																	
Randomisation		×																
Compliance check for Life style instructions & drug		×														×	×	×
AE check and change in concomitant medication		×														×	×	×

Dosing period: Day 1 to Day 28

Hepatic MRS will be done on Day 0 and Day 28.(A deviation of ±2 days is acceptable)

These are exploratory investigations and the exact list will be determined based on clamp study findings. Till then, samples will be preserved.

APPENDIX C: Flow Chart of Clamp Procedure:

App Time	Day	Duration/ T=	Activity
20:00	-1 and 27	13 hr	Fasting
07:30	0 and 28	30 min	MRS
08:00	0 and 28	-2hr	Two catheters will be inserted in both the arms. Right side will be used for infusion and Left side will be used for blood sample collection
09:00	0 and 28	-2 hr	• Blood drawing for background enrichment of plasma glucose<br>• Priming dose: equivalent to 100 min of infusion at rate 0.11 µmol/kg per min<br>• A continuous infusion of [6,6-2H2]-glucose (>99% enriched, Cambridge Isotopes, Massachusetts, USA) is started at a rate of 0.11 µmol/kg per min till the end of clamp (6:20hr).<br>• Priming dose: 1.6 µmol/kg/min. A continuous infusion of [1,1,2,3,3-2H2]-glycerol (>99% enriched, Cambridge Isotopes, Massachusetts, USA) is started at a rate of 0.11 µmol/kg per min till the end of clamp (5:20hr).
10:50	0 and 28	-0:10	Blood sample will be collected for determination of<br>• Glucose and glycerol enrichment<br>• Glucoregulatory hormones (Insulin, Glucagon & Catecholamine)<br>• FFA
10:55	0 and 28	-0:05	
11:00	0 and 28	0:00	
11:00	0 and 28	0:00	The hyperinsulinemic clamp will start with a continuous infusion of insulin (Actrapid 100U/ml, Novo Nordisk) for 2h at a rate of 20mU/m2 body surface area per min.
11:00-13:05 BG will be estimated at every 10 min	0 and 28	0:00-2:05	Plasma glucose is measured every 10 min (via Biosen/YSI analyzer) and glucose 20% is infused at a variable rate to maintain plasma glucose at 5.0 mmol/l. [6,6-2H2]-glucose is added to the 20% glucose solution to achieve glucose enrichments of 1% to minimize changes in isotopic enrichment due to changes in the infusion rate of exogenous glucose, and thus to allow for accurate quantification of endogenous glucose production and uptake
12:50	0 and 28	1:50	Blood sample will be collected for determination of<br>• Glucose and glycerol enrichment<br>• Glucoregulatory hormones Insulin, Glucagon & Catecholamine)<br>• FFA
12:55		1:55	
13:00		2:00	
13:05		2:05	
13:10		2:10	
13:15– 15:15	0 and 28	2:15-4:15	The infusion of insulin (Actrapid 100U/ml, Novo Nordisk) is increased to a rate of 60mU/m2 body surface area per min.
13:15-15:15 BG will be	0 and 28	2:15-4:15	Plasma glucose is measured every 10 min (via Biosen/YSI analyzer) and glucose 20% is infused at a variable rate to

estimated at every 10 min			maintain plasma glucose at 5.0 mmol/l. [6,6-2H2]-glucose is added to the 20% glucose solution to achieve glucose enrichments of 1% to minimize changes in isotopic enrichment due to changes in the infusion rate of exogenous glucose, and thus to allow for accurate quantification of endogenous glucose production and uptake
15:00	0 and 28	4:00	During the last 20 min of this insulin infusion step, blood samples are drawn at 5 min intervals for determination of<br>• Glucose and glycerol enrichment,<br>• Glucoregulatory hormones<br>• FFA
15:05		4:05	
15:10		4:10	
15:15		4:15	
15:20		4:20	
15:20	0 and 28	4:20	Stop insulin infusion & Double the glucose infusion rate
15:20	0 and 28	4:20	Provide a carbohydrate rich meal to volunteer.
15:20 onwards BG will be estimated at every 10 min	0 and 28	4:30	If plasma glucose reading in > 7 mmol/l (>126 mg/dl), then reduce the glucose infusion rate to half, until it becomes less than 5 ml/hr.

14. REFERENCES

1. Després JP, Lemieux I, Bergeron J, et al. Abdominal Obesity and the Metabolic Syndrome: Contribution to Global Cardiometabolic Risk. Arterioscler. Thromb. Vasc. Biol. 2008;28:1039-1049.
2. Grundy SM, Cleeman JI, Daniels SR, et al. Diagnosis and Management of the Metabolic Syndrome: An American Heart Association/National Heart, Lung, and Blood Institute Scientific Statement: Executive Summary. Circulation 2005,112:2735-2752.
3. Simone GD, Devereux RB, Chinali M, et al. Prognostic Impact of Metabolic Syndrome by Different Definitions in a Population with High Prevalence of Obesity and Diabetes. Diabetes Care 2007,30:1851–1856.
4. Chen K, Lindsey JB, Khera A, et al. Independent associations between metabolic syndrome, diabetes mellitus and atherosclerosis: observations from the Dallas Heart Study Diabetes. Vasc Dis Res 2008,5:96–101
5. Enas AE, Vishwanathan M, Mohan D, et al. The Metabolic Syndrome and Dyslipidemia Among Asian Indians: A Population With High Rates of Diabetes and Premature Coronary Artery Disease. J Cardiometab Syndr. 2007,2(4):267-275.
6. Lorenzo C, Stern MP, Okoloise M, et al. The Metabolic Syndrome as Predictor of Type 2 Diabetes. The San Antonio Heart Study. Diabetes Care 2003,26:3153- 3159.
7. Shimabukuro M. Cardiac Adiposity and Global Cardiometabolic Risk: New Concept and Clinical Implication. Circ J 2009;73:27 – 34
8. Wilson PWF, Meigs JB. Cardiometabolic risk: a Framingham perspective. International Journal of Obesity 2008,32:S17-S20
9. Koves TR, Ussher JR, Noland RC et al. Mitochondrial overload and incomplete fatty acid oxidation contribute to skeletal muscle insulin resistance. Cell Metab 2008;7(1):45-56.
10. Bays H et al Adiposopathy: why do adiposity and obesity cause metabolic disease? Future Lipidol. 2006,1:389-420.
11. Nisoli et al. Defective mitochondrial biogenesis: a hallmark of the high cardiovascular risk in the metabolic syndrome? Circulation Research 2007,100:795-806.
12. Finocchietto P et al. Control of muscle mitochondria by insulin entails activation of Akt2-mtNOS pathway: implications for the metabolic syndrome. PLoS ONE 2008,3:e1749.
13. Gougeon R et al. The prediction of resting energy expenditure in type-2 diabetes mellitus is improved by factoring for glycemia. Int J Obes Relat Metab Disord. 2002,26:1547-1552.
14. Simoneau JA et al. Markers of capacity to utilize fatty acids in human skeletal muscle: relation to insulin resistance and obesity and effects of weight loss. FASEB J. 1999,13:2051-2060.
15. Kelley DE et al. Dysfunction of mitochondria in human skeletal muscle in type-2 diabetes. Diabetes 2002, 51:2944-2950.
16. Song J et al. Peripheral blood mitochondrial DNA content is related to insulin sensitivity in offspring of type-2 diabetic patients. Diabetes Care 2001,24:865-869.

17. Patti ME. Gene expression in humans with diabetes and prediabetes: what have we learned about diabetes pathophysiology? Curr Opin Clin Nutr Metab Care 2004, 7:383-390.
18. Petersen KF et al. Impaired mitochondrial activity in the insulin-resistant offspring of patients with type-2 diabetes. N Engl J Med 2004, 350:664-671.
19. Arbeeny CM. Addressing the Unmet Medical Need for Safe and Effective Weight Loss Therapies. Obes Res. 2004,12:1191-1196.
20. Salter WT, Lerman J, Means JH. The calorigenic action of thyroxin polypeptide. J Clin Invest 1933;12(2):327-334.
21. Aronne LJ, Thornton-Jones ZD. New targets for obesity pharmacotherapy. Clin Pharmacol Ther 2007;81(5):748-752.
22. Ladenson PW, Kristensen JD, Ridgway EC et al. Use of the thyroid hormone analogue eprotirome in statin-treated dyslipidemia. N Engl J Med 2010;362(10):906-916.
23. Wexler JA, Sharretts J. Thyroid and bone. Endocrinol Metab Clin North Am 2007;36(3):673-705, vi.
24. Biondi B, Cooper DS. The clinical significance of subclinical thyroid dysfunction. Endocr Rev 2008;29(1):76-131.
25. Moreno M, de LP, Lombardi A, Silvestri E, Lanni A, Goglia F. Metabolic effects of thyroid hormone derivatives. Thyroid 2008;18(2):239-253.
26. Cioffi F, Zambad SP, Chhipa L, et al. TRC150094, a novel functional analog of iodothyronines, reduces adiposity by increasing energy expenditure and fatty acid oxidation in rats receiving a high-fat diet. FASEB J 2010;24:3451-3461
27. Alberti KGMM, Eckel RH, Grundy SM, et al. Harmonizing the Metabolic Syndrome: A Joint Interim Statement of the International Diabetes Federation Task Force on Epidemiology and Prevention; National Heart, Lung, and Blood Institute; American Heart Association; World Heart Federation; International Atherosclerosis Society; and International Association for the Study of Obesity. Circulation 2009;120:1640-1645.
28. Steele R. Influences of glucose loading and of injected insulin on hepatic glucose output. Ann NY Acad Sci 1959;82:420-430.
29. Riserus U, Zethelius B, Arnlov J, et al. Sagittal Abdominal Diameter Is a Strong Anthropometric Marker of Insulin Resistance and Hyperproinsulinemia in Obese Men. Diabetes Care 2004;27:2041-2046
